# Collagen I-induced VCAN/ERK signaling and PARP1/ZEB1-mediated metastasis facilitate OSBPL2 defect to promote colorectal cancer progression

**DOI:** 10.1038/s41419-024-06468-1

**Published:** 2024-01-24

**Authors:** Kang Lin, Yun Zhao, Yuqi Tang, Ying Chen, Moubin Lin, Luwei He

**Affiliations:** 1 Center for Clinical Research and Translational Medicine, Yangpu Hospital, School of Medicine, Tongji University, Shanghai, China; 2 Institute of Gastrointestinal Surgery and Translational Medicine, School of Medicine, Tongji University, Shanghai, China; 3 State Key Laboratory of Cell Biology, Shanghai Institute of Biochemistry and Cell Biology, Center for Excellence in Molecular Cell Science, Chinese Academy of Sciences; University of Chinese Academy of Sciences, Shanghai, China; 4 School of Life Science and Technology, ShanghaiTech University, Shanghai, China; 5 Key Laboratory of Systems Health Science of Zhejiang Province, School of Life Science, Hangzhou Institute for Advanced Study, University of Chinese Academy of Sciences, Hangzhou, China; 6 Department of General Surgery, Yangpu Hospital, School of Medicine, Tongji University, Shanghai, China

**Keywords:** Cell growth, Extracellular matrix, Cell migration, Targeted therapies, Tumour biomarkers

## Abstract

The global burden of colorectal cancer (CRC) has rapidly increased in recent years. Dysregulated cholesterol homeostasis facilitated by extracellular matrix (ECM) remodeling transforms the tumor microenvironment. Collagen I, a major with ECM component is highly expressed in colorectal tumors with infiltrative growth. Although oxysterol binding protein (OSBP)-related proteins accommodate tumorigenesis, OSBPL2, which is usually involved in deafness, is not associated with CRC progression. Therefore, we aimed to investigate the pathological function of OSBPL2 and identify the molecular link between ECM-Collagen I and OSBPL2 in CRC to facilitate the development of new treatments for CRC. OSBPL2 predicted a favorable prognosis in stage IV CRC and substantially repressed Collagen I-induced focal adhesion, migration, and invasion. The reduction of OSBPL2 activated ERK signaling through the VCAN/AREG/EREG axis during CRC growth, while relying on PARP1 via ZEB1 in CRC metastasis. OSBPL2 defect supported colorectal tumor growth and metastasis, which were suppressed by the ERK and PARP1 inhibitors SCH772984 and AG14361, respectively. Overall, our findings revealed that the Collagen I-induced loss of OSBPL2 aggravates CRC progression through VCAN-mediated ERK signaling and the PARP1/ZEB1 axis. This demonstrates that SCH772984 and AG14361 are reciprocally connective therapies for OSBPL2^Low^ CRC, which could contribute to further development of targeted CRC treatment.

## Introduction

Colorectal cancer (CRC) is a prevalent malignancy worldwide, with a high incidence and mortality [1]. Population aging has increased the CRC burden in China and the United States of America. This warrants further development of strategies for cancer prevention and treatment [2].

Oxysterol-binding protein (OSBP)-related proteins (ORPs) are lipid transfer proteins that exchange phosphoinositide phosphatidylinositol 4-phosphate for cholesterol between adjacent membranes. They control cell signaling and vesicle transport [3–8]. Increased cholesterol influx and synthesis, as well as decreased efflux, alter the tumor microenvironment (TME). Additionally, these changes alter sensitivity to chemotherapy and radiation treatment and contribute to metastasis [9–11]. The tumor extracellular matrix (ECM) is a core component of the TME. ECM remodeling affects cancer cell signaling, proliferation, and metastasis [12, 13]. The components of ECM comprise two major classes, namely, collagens and glycoproteins [14]. Collagen I is highly expressed in colorectal tumors with infiltrative growth [15]. Versican, a chondroitin sulfate proteoglycan, is also linked to malignant transformation and tumor progression [16]. Thus, Collagen I and Versican are potential TME markers and targets for CRC therapy [17].

ORP3, -4, -5 and -8 can regulate tumorigenesis, whereas ORP2 gains copy numbers in invasive CRC [18, 19]. ORP2 (OSBPL2) comprises an OSBP homology domain and two phenylalanine molecules in an acidic tract. This facilitates cholesterol efflux, cortisol production, and lipid droplet dispersion [20–22]. OSBPL2 binds to COPB1 and mediates the transport of the lipolytic lipase PNPLA from the endoplasmic reticulum (ER) to lipid droplets [23]. Furthermore, whole-exome sequencing has linked this protein to deafness, owing to primary cilia defects and abnormal Sonic Hedgehog signal transduction [24–27]. The hearing loss caused by OSBPL2 mutation has been attributed to reciprocal reverse regulation of OSBPL2 and 25-hydroxycholesterol (25-OHC) on ER-Golgi sites. It has also been attributed to the loss of interaction between OSBPL2 and the Ras homolog A (RhoA) effector diaphanous homolog 1 [28, 29]. OSBPL2 deletion upregulates sterol biosynthesis and reactive oxygen species (ROS) levels by suppressing AMPK activity in auditory cells [30, 31]. In addition, accumulation of OSBPL2 mutations leads to dysfunctional autophagy and hearing loss, which are partially remedied by rapamycin [32].

OSBPL2 regulates the actin cytoskeleton to form lamellipodia through RhoA and AKT signaling, thereby affecting hepatocellular energy metabolism, migration, adhesion, and proliferation [33–35]. It transfers LDL-derived cholesterol from late to recycling endosomes for FAK (Focal Adhesion Kinase) activation and cell adhesion in human epithelial carcinoma cells [36]. In endothelial cells, this protein is associated with angiogenic signaling and angiogenesis *via* VEGFR2 [37]. However, the association between OSBPL2 expression and CRC progression remains unclear. Therefore, we systematically research the pathological function and molecular mechanism of OSBPL2 with Collagen-I in CRC development and therapies of ERK and PARP1 inhibitors for OSBPL2 loss. Our findings could substantially contribute to the identification of treatment targets to facilitate effective development of CRC treatment at various stages.

## Results

### OSBPL2 predicts a favorable prognosis in patients with Stage IV CRC

**Fig. 1:**
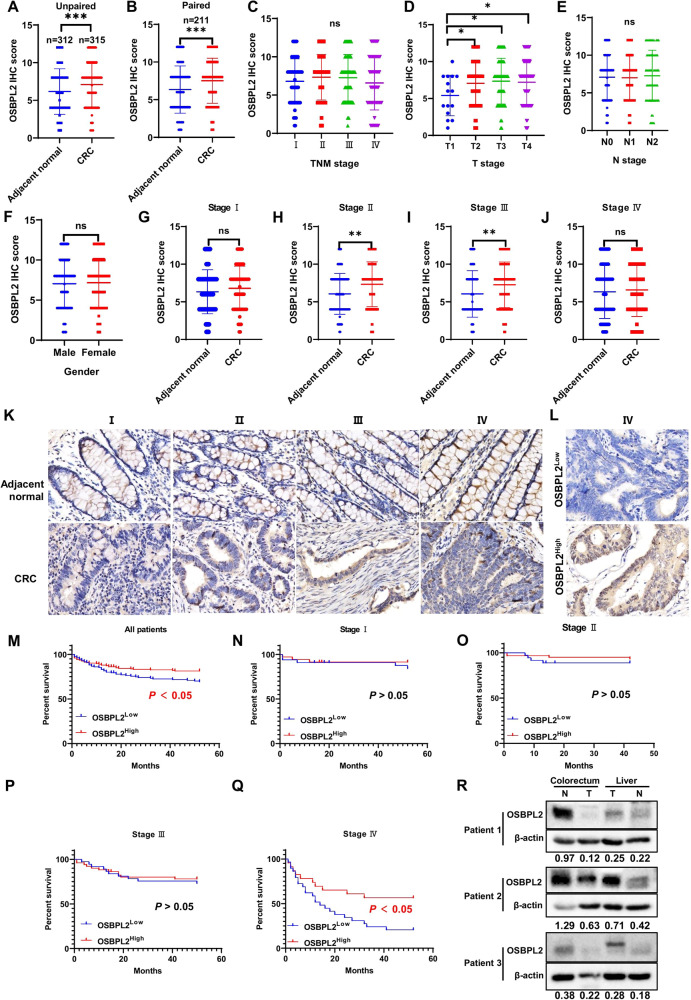
OSBPL2 predicts favorable prognosis in Stage IV CRC patients. **A**–**J** IHC score of OSBPL2 in unpaired (**A**) and paired (**B**) adjacent normal and CRC tumor tissues. OSBPL2 expression in different TNM stage (**C**), T stage (**D**), N stage (**E**). IHC score of OSBPL2 in adjacent normal and CRC tumor tissues with stage I (**F**), stage II (**G**), stage III (**H**) and stage IV (**I**). OSBPL2 expression in different gender (**J**). **K** OSBPL2 expression in adjacent normal and CRC tumor tissues by IHC analysis. **L** OSBPL2 expression (OSBPL2^Low^ and OSBPL2^High^) in stage IV CRC tumor tissues by IHC analysis. Score 0–7 assigned to the low expression level, while score 8–12 assigned to the high expression level. Scale bars, 10 μm. **M**–**Q** Kaplan–Meier survival analysis of overall survival in all patients (**M**), stage I (**N**), stage II (**O**), stage III (**P**) and stage IV (**Q**) patients with OSBPL2^Low^ expression and OSBPL2^High^ expression. **R** The expression of OSBPL2 in primary tumors and liver metastases of patients. The ratio of SQLE/β-actin was labelled in below. N: adjacent normal, T: tumor. Mean ± SD. *P < 0.05; **P < 0.01; ***P < 0.001; ns no significance.

To explore the pathological function of OSBPL2 in CRC, we collected 312 normal and 315 CRC clinical samples for TMA analysis. In unpaired and paired CRC samples, the IHC score of OSBPL2 was remarkably elevated in CRC tissues compared to normal tissues (Fig. 1A, B). Similar results were observed in pan-cancer and CRC cell lines (Fig. S1A–S1B). However, the levels of OSBPL2 in TNM stages I, II, III, and IV showed no significant differences (Fig. 1C). Although OSBPL2 expression was substantially higher in T2/T3/T4 than in T1 (Fig. 1D), it did not correlate with N stage or gender (Fig. 1E, F). Moreover, IHC staining of OSBPL2 showed no differences between CRC tissues and adjacent normal tissues in stages I and IV. In contrast, there were statistically significant differences in stages II and III (Fig. 1G–J). Representative IHC images are shown in Figs. 1K, L. The Kaplan–Meier and log-rank tests revealed that patients with CRC characterized by lower OSBPL2 levels had poorer overall survival in all stages and stage IV (Fig. 1M–Q). Similarly, survival analysis in the TCGA database showed that the downregulation of OSBPL2 expression was a malignant factor in patients with metastatic CRC (Fig. S1C–G). In addition, OSBPL2 expression did not increase in stages IV, T4, or N2 in the TCGA cohort (Fig. S1H–J). The level of OSBPL2 was also not associated with the age and gender of patients with CRC in the TCGA cohort (Figure S1K–L). Additionally, OSBPL2 levels decreased in the primary colorectal tumors and liver metastases of patients with stage IV CRC, contrary to normal colorectal tissues (Fig. 1R). These results indicate that OSBPL2 may be regulated by the dynamic TME and may have protective properties against metastatic CRC.

### OSBPL2 promotes CRC cell proliferation in vitro without extracellular matrix

**Fig. 2:**
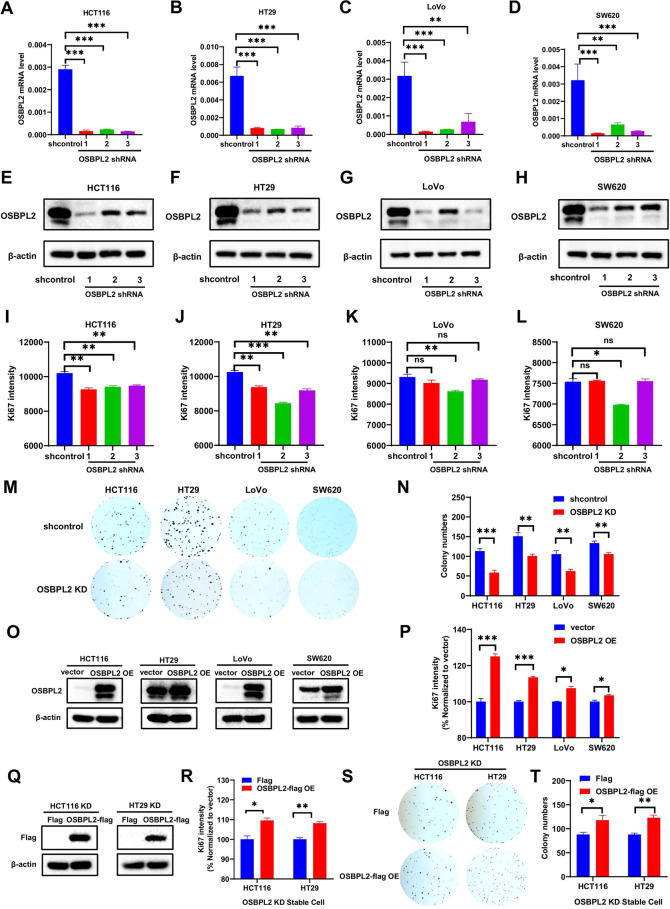
OSBPL2 promotes CRC cell proliferation without extracellular matrix in vitro. After utilizing three shRNAs of OSBPL2, qPCR analysis was performed to measure the mRNA expression levels of OSBPL2 in HCT116 (**A**), HT29 (**B**), LoVo (**C**), and SW620 (**D**) cells, shcontrol was served as control. Mean ± SD. Protein level of OSBPL2 was analyzed by western blotting in HCT116 (**E**), HT29 (**F**), LoVo (**G**), and SW620 (**H**) cells, following knockdown of OSBPL2, shcontrol was served as control. Flow cytometry was used to analyze ki67 intensity in HCT116 (**I**), HT29 (**J**), LoVo (**K**), and SW620 (**L**) cells after knockdown of OSBPL2, shcontrol was served as control. **M**, **N** Colony formation assays were performed on HCT116 (NO.1 shRNA), HT29 (NO.3 shRNA), LoVo (NO.2 shRNA), and SW620 (NO.2 shRNA) cells after knockdown of OSBPL2 (OSBPL2 KD), shcontrol was served as control. **O** Western blotting was performed to measure protein level of OSBPL2 in HCT116, HT29, LoVo, and SW620 cells after overexpression of OSBPL2 (OSBPL2 OE), vector was served as control. **P** After overexpression of OSBPL2, ki67 intensity was analyzed in HCT116, HT29, LoVo, and SW620 cells by flow cytometry, vector was served as control. **Q** Flag-tagged OSBPL2 was overexpressed in OSBPL2 KD stable HCT116 and HT29 cell lines, Flag protein level was analyzed by western blotting, vector was served as control. **R** Ki67 intensity was analyzed by flow cytometry in OSBPL2 KD stable HCT116 and HT29 cell lines overexpressing Flag-tagged OSBPL2, vector was served as control. **S**, **T** Colony formation assays were performed on OSBPL2 KD stable HCT116 and HT29 cell lines overexpressing Flag-tagged OSBPL2. β-actin was used as loading control. Mean ± SEM. *P < 0.05; **P < 0.01; ***P < 0.001; ns no significance.

To investigate the biological function of OSBPL2 in CRC, we constructed stable OSBPL2 knockdown (KD) cell lines via shRNA interference in HCT116, HT29, LoVo, and SW620 cells. We further verified the knockdown efficiency using real-time PCR and western blotting (Fig. 2A–H). We used flow cytometry (FACS) to identify the impact of OSBPL2 knockdown on CRC cell growth, revealing that Ki67 levels were reduced to various degrees (Figs. 2I–L and S2). OSBPL2 knockdown markedly inhibited colony formation in HCT116, HT29, LoVo, and SW620 cells (Fig. 2M, N). Moreover, we overexpressed OSBPL2 (OSBPL2 OE) in HCT116, HT29, LoVo, and SW620 cells to further investigate the function of OSBPL2 in CRC cell growth (Fig. 2O). FACS analysis showed that OSBPL2 OE markedly increased Ki67 expression (Figs. 2P and S2). Similarly, we constructed stable OSBPL2-overexpressed (OSBPL2-flag OE) HCT116 and HT29 KD cell lines to perform rescue experiments (Fig. 2Q). Overexpression of OSBPL2 in HCT116 and HT29 KD cells expedited their growth and colony formation (Fig. 2R–T). These data show that OSBPL2 accelerates CRC cell proliferation without extracellular matrix by elevating Ki67 levels.

### Collagen I renders loss of OSBPL2 contribute to cell growth, focal adhesion and invadopodia formation

**Fig. 3:**
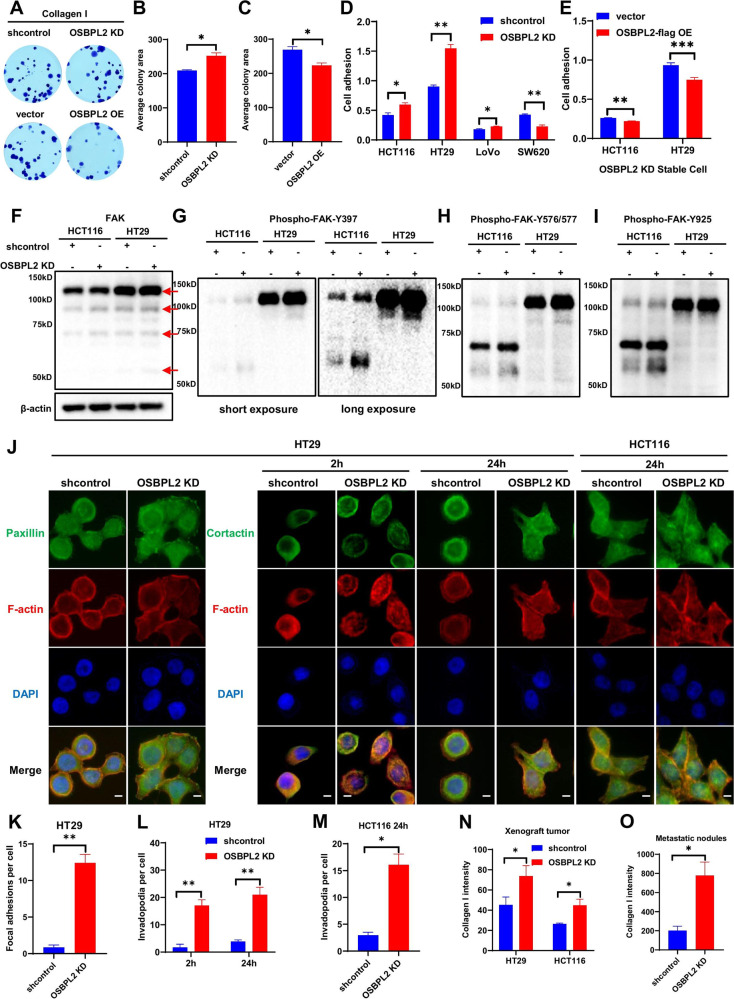
Collagen I renders OSBPL2 defect contribute to cell growth, focal adhesion and invadopodia formation. **A**–**C** Colony formation assays were performed on OSBPL2 KD and OE stable HCT116 cell lines with the incubation of Collagen I. **D**, **E** The Collagen I-mediated cell adhesion assay was performed on shcontrol and OSBPL2 KD of HCT116, HT29, LoVO and SW620 cells, as well as on vector and OSBPL2-flag OE of OSBPL2 KD HCT116 and HT29 cells. The protein levels of total FAK (**F**), Phospho-FAK-Y397 (**G**), Phospho-FAK-Y576/577 (**H**), and Phospho-FAK-Y925 (**I**) were assessed in shcontrol and OSBPL2 KD HCT116 and HT29 cells by western blotting. β-actin was served as a loading control. The bands of FAK were marked in red arrows. **J**–**M** Immunofluorescence was performed to detect focal adhesion and invadopodia in shcontrol and OSBPL2 KD HT29 cells with stimulation of Collagen I for 2 h or 24 h, as well as in shcontrol and OSBPL2 KD HCT116 cells for 24 h. Paxillin and Cortactin were labelled in green, F-actin was labelled in red, and the cell nucleus were stained by DAPI (in blue). Scale bars, 10 μm. **N**, **O** IHC analysis of Collagen I expression in Figs. 6A, 8A. Scale bars, 20 μm.

To further understand biological function of OSBPL2 on connection between cell and extracellular matrix (ECM), we applied Collagen I treated with CRC cells. Decreased OSBPL2 levels following Collagen I treatment stimulated the growth of CRC cells. Contrastingly, overexpression of OSBPL2 alleviated the growth of CRC cells (Fig. 3A–C). OSBPL2 knockdown increased the adhesion of HCT116, HT29, and LoVo cells, but not that of SW620 cells (Fig. 3D). In contrast, OSBPL2 overexpression in stable OSBPL2 KD cells decreased cell adhesion (Fig. 3E). Detection of the FAK signaling pathway involved in cell adhesion revealed that reduced OSBPL2 expression elevated the phosphorylation of FAK more at Y397 than at Y576/577 and Y925 (Fig. 3F–I). The crosslinking and stiffness of the ECM cause protrusive invadopodia with proteolytic activity and invasive capabilities [38]. Therefore, CRC cells were stained with F-actin, Paxillin, and Cortactin to directly visualize focal adhesions and invadopodia induced by Collagen I. Decreased OSBPL2 expression improved focal adhesion and invadopodia in HT29 and HCT116 cells (Fig. 3J–M). Otherwise, the level of Collagen I was evaluated based on the OSBPL2 defect in the in vivo model of colorectal tumor progression (Figs. 3N–O and S3). These findings indicate that OSBPL2 inhibits CRC cell growth, adhesion, and invasion with the assistance of Collagen I.

### OSBPL2 deficiency accelerates CRC cell migration and invasion

**Fig. 4:**
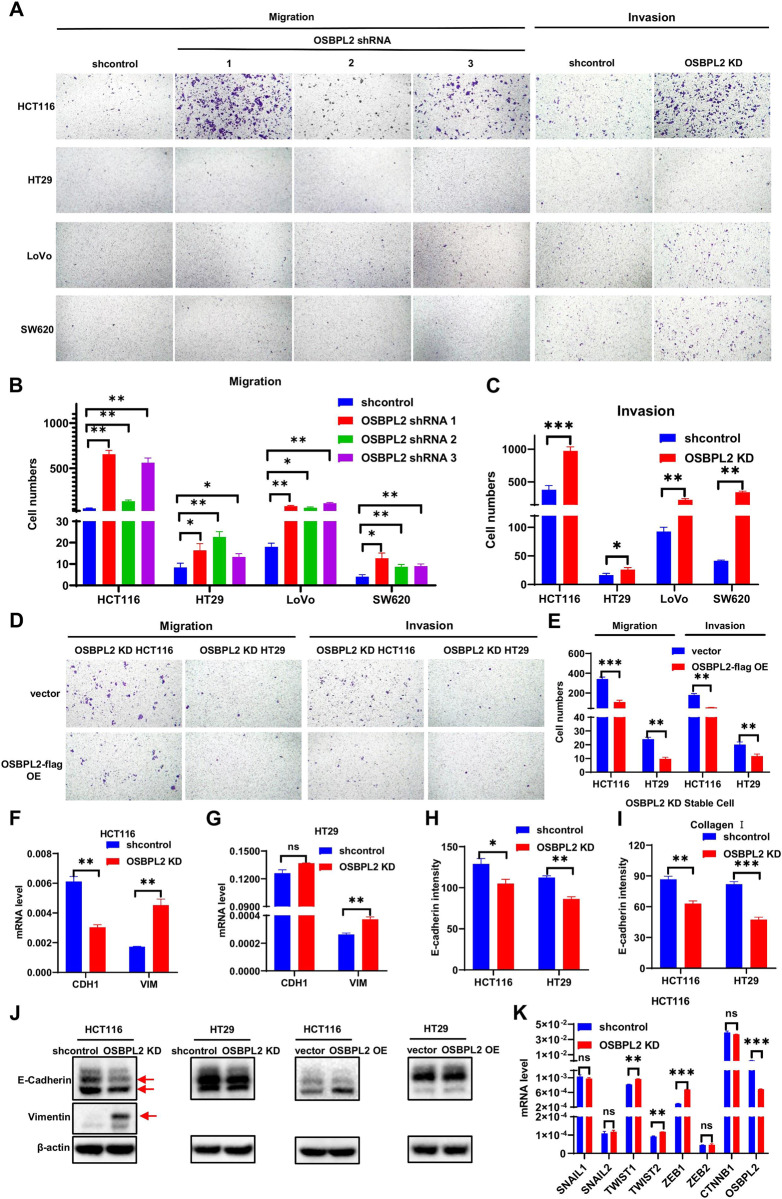
OSBPL2 deficiency accelerates migration and invasion of CRC cells. **A** Transwell migration assay and invasion assay for shcontrol and OSBPL2 KD HCT116, HT29, LoVo and SW620, shcontrol was served as control. **B**, **C**. The statistic data of (**A**). **D** Transwell migration assay and invasion assay for vector and overexpression of OSBPL2-flag in OSBPL2 KD HCT116 and HT29 cells, vector was served as control. **E** The statistic data of (**D**). **F**, **G** Real-time PCR was utilized to assess the mRNA level of CDH1, VIM, and TWIST1 in shcontrol and OSBPL2 KD HCT116, HT29 cells, ACTB was used as a housekeeping gene. **H**, **I** Immunofluorescence staining was employed to analyze the intensity of E-cadherin in shcontrol and OSBPL2 KD HCT116 and HT29 cells with or without Collagen I. **J** Western blotting was employed to detect the protein levels of E-cadherin and Vimentin in shcontrol and OSBPL2 KD HCT116, HT29 cells, as well as in vector and OSBPL2 OE HCT116, HT29 cells. β-actin was utilized as the loading control. The bands of E-cadherin and Vimentin were marked in red arrows. **K** Real-time PCR was utilized to assess the mRNA expression of SNAIL1, SNAIL2 TWIST1, TWIST2, ZEB1, ZEB2, CTNNB1 and OSBPL2 in shcontrol and OSBPL2 KD HCT116 cells, shcontrol was used as a control. Mean ± SEM. *P < 0.05; **P < 0.01; ***P < 0.001; ns no significance.

A transwell assay was used to test the migration and invasion capacities of CRC cells to assess the effect of OSBPL2 on CRC metastasis. OSBPL2 deficiency substantially promoted CRC cell migration and invasion (Fig. 4A–C), whereas OSBPL2 overexpression in HCT116 and HT29 cells inhibited these processes (Fig. 4D, E). To further assess the alleviated cell migration and invasion induced by OSBPL2, we evaluated the EMT process that was hijacked to induce changes in morphology and motility [39]. EMT is defined as the disappearance of the epithelial marker E-cadherin and occurrence of the mesenchymal marker vimentin [40]. OSBPL2 knockdown markedly reduced E-cadherin expression and enhanced vimentin expression in HCT116 cells (Fig. 4F, G). Immunofluorescence staining confirmed that OSBPL2 defect suppressed the level of E-cadherin in CRC cells, with or without Collagen I treatment (Fig. 4H, I, S4). Additionally, decreased OSBPL2 expression downregulated E-cadherin and upregulated vimentin protein levels (Fig. 4J). The protein level of E-cadherin was slightly upregulated, and Vimentin was hardly detectable following OSBPL2 overexpression (Fig. 4J). Low vimentin levels were detected, owing to rare gene expression in HT29 cells (Fig. 4J). In addition, OSBPL2 knockdown markedly enhanced the expression of Twist1, Twist2, and ZEB1, which are key transcription factors involved in EMT regulation (Fig. 4K). These results indicate that OSBPL2 defect facilitates CRC cell migration and invasion *via* EMT.

### OSBPL2 modifies ERK signaling through the VCAN/AREG/EREG axis in Collagen I-induced cell growth but not in EMT

**Fig. 5:**
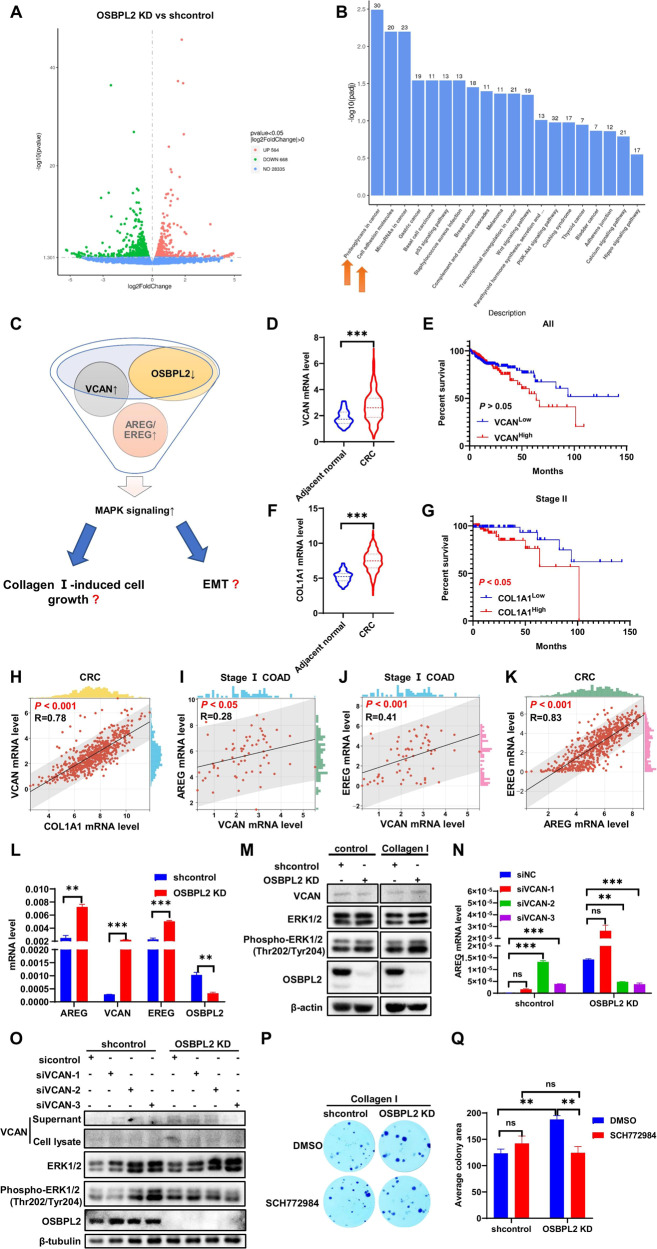
OSBPL2 modifies ERK signaling through VCAN/AREG/EREG axis in Collagen I-induced cell growth not in EMT. **A** The volcano plots of genes in shcontrol and OSBPL2 KD HCT116 cells analyzed by RNA-seq. **B** The KEGG enrichment pathways of differential genes followed by knockdown of OSBPL2. **C** The hypothesis of regulation of VCAN/AREG/EREG by OSBPL2 deficiency. **D**–**G** The mRNA expression of VCAN (**D**) and COL1A1 (**F**) in CRC tumor tissues and normal tissues (from TCGA database). Tumor samples in COAD were divided into high and low expression groups based on the median VCAN or COL1A1 expression level, and the survival of the two groups was analyzed using the Kaplan–Meier method in all stages (**E**) and stage II (**G**). **H** The correlation between COL1A1 and VCAN in CRC. **I** The correlation between VCAN and AREG in Stage I COAD (Colorectal adenocarcinoma). **J** The correlation between VCAN and EREG in Stage I COAD. **K** The correlation between AREG and EREG in CRC. **L** The expressions of VCAN/AREG/EREG were examined by real-time PCR in shcontrol and OSBPL2 KD HCT116 cells, ACTB was used as a housekeeping gene. **M** The protein levels of total ERK and Phospho-ERK1/2 (Thr202/Tyr204) in shcontrol and OSBPL2 KD HCT116 cells were measured by western blot. **N** After knockdown Versican by three VCAN siRNAs, the expressions of AREG were examined by real-time PCR, ACTB was used as a housekeeping gene. **O** The protein levels of total ERK and Phospho-ERK1/2 (Thr202/Tyr204) were measured by western blot, followed by knockdown Versican by three VCAN siRNAs. **P**, **Q** The shcontrol and OSBPL2 KD HCT116 were treated with SCH772984 (1.25 nM), respectively, then be detected by colony formation assay.

Transcriptome sequencing was performed to investigate the molecular mechanisms underlying OSBPL2 expression in CRC. RNA-seq analysis showed 564 and 668 genes whose expression was upregulated and downregulated, respectively, in OSBPL2 KD HCT116 cells compared to shcontrol cells (Fig. 5A). Proteoglycans in cancer and cell adhesion molecules were the two most enriched Kyoto Encyclopedia of Genes and Genomes (KEGG) pathways (Fig. 5B). The two most differentially expressed genes were amphiregulin (AREG) and Versican (VCAN) (Table S1). AREG, a member of the epidermal growth factor (EGF) family, regulates mitogen-activated protein kinase (MAPK) signaling. The expression of epiregulin (EREG), another EGF, was upregulated by OSBPL2 knockdown.

VCAN is a cell adhesion molecule in the ECM that coordinates with EGF to modify MAPK signaling in OSBPL2 KD CRC cells (Fig. 5C). In the TCGA database, the expression of VCAN and COL1A1 was elevated in CRC. In addition, higher COL1A1 levels were associated with poor prognosis in stage II CRC (Fig. 5D–G). Although VCAN was not a prognostic marker in CRC, it positively correlated with COL1A1 (Fig. 5H). Additionally, VCAN correlated positively with AREG and EREG in stage I, and AREG correlated positively with EREG in all stages (Fig. 5I–K). Thus, the expression of AREG, EREG, and VCAN and the activation of MAPK signaling were examined. AREG, EREG, and VCAN levels were enhanced by OSBPL2 knockdown (Fig. 5L). Additionally, OSBPL2 deficiency stimulated ERK phosphorylation and VCAN accumulation following incubation with Collagen I (Fig. 5M). VACN knockdown by effective siRNA-2, 3 eliminated the phosphorylation of ERK and increased AREG levels in OSBPL2 KD CRC cells (Figs. 5N–O and S5A). Furthermore, the ERK inhibitor SCH772984 impaired Collagen I-induced colony formation, but not invasion. This was followed by the loss of OSBPL2 (Figs. 5P, Q and S5B, C). These findings demonstrate that a decrease in OSBPL2 provokes VCAN/AREG/AREG-mediated ERK signaling during CRC cell growth.

### OSBPL2 protects against colorectal tumor growth by blocking ERK phosphorylation

**Fig. 6:**
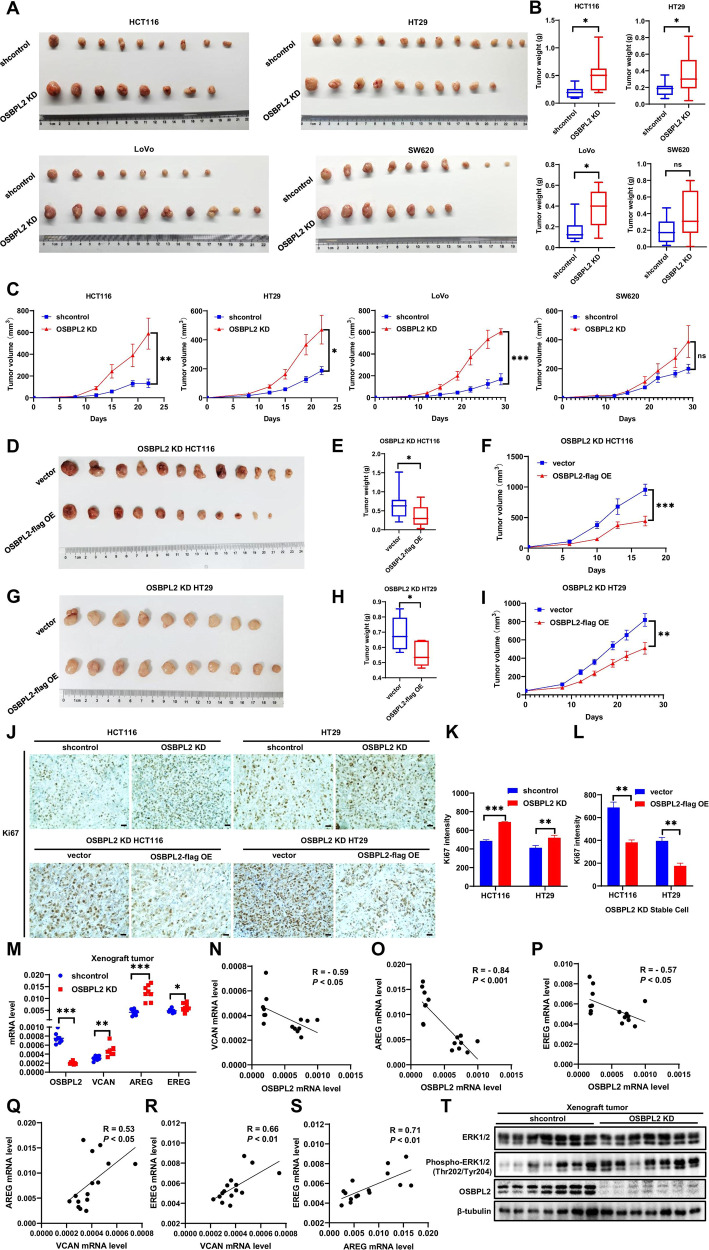
OSBPL2 protects against colorectal tumor growth *via* blocking ERK phosphorylation. Images (**A**) and weights (**B**) of the tumors harvested from nude mice and tumor growth curve (**C**) of shcontrol and OSBPL2 KD HCT116 (*n* = 11), HT29 (*n* = 11), LoVo (*n* = 11) and SW620 (*n* = 10) cells. Images (**D**, **G**) and weights (**E**, **H**) of the tumors harvested from nude mice and tumor growth curve (**F**, **I**) of vector and overexpression of OSBPL2-flag in OSBPL2 KD HCT116 (*n* = 12) and HT29 (*n* = 10) cells. **J**–**L** IHC analysis of Ki67 expression in (**A**), (**D**) and (**G**). Scale bars, 20μm. M. The expressions of OSBPL2/VCAN/AREG/EREG were examined by real-time PCR in xenograft tumors derived by shcontrol and OSBPL2 KD HCT116 cells. **N**–**S** The correlation among OSBPL2, VCAN, AREG, EREG in (**M**) were measured by linear regression and Pearson analysis. **T** The protein levels of total ERK, Phospho-Erk1/2 (Thr202/Tyr204) and OSBPL2 in xenograft tumors were detected by western blot, β-tubulin was utilized as the loading control. Mean ± SEM. *P < 0.05; **P < 0.01; ***P < 0.001. ns no significance.

We used a xenograft tumor model to assess the function of OSBPL2 in colorectal tumor growth in vivo. As shown in Fig. 6A–C, OSBPL2 deficiency significantly expedited the growth of tumors derived from CRC cells, except for SW620 cells. The average weights and volumes of xenograft tumors in mice injected with shcontrol HCT116, HT29, and LoVo cells reached 0.19 g, 0.18 g, and 0.16 g and 149.53 mm^3^, 187.18 mm^3^, and 199.83 mm^3^, respectively. The average weight and tumor volume of mice injected with OSBPL2 KD CRC cells increased by 169.41%, 100.48%, and 129.24% and 294.50%, 151.24%, and 202.70%, respectively. In contrast, OSBPL2 overexpression reduced the weights and volumes of HCT116 and HT29 cell-derived xenograft tumors by 43.18%, 20.62% and 53.58%, 37.69%, respectively (Fig. 6D–I). Consistent with these observations, Ki67 expression considerably increased after the downregulation of OSBPL2, whereas the upregulation of OSBPL2 markedly decreased Ki67 expression (Fig. 6J–L). The expression levels of VCAN, AREG, and EREG were evaluated in tumors derived from OSBPL2 KD CRC cells (Fig. 6M). OSBPL2 was negatively correlated with VCAN, AREG, and EREG, whereas there was a positive correlation between VCAN, AREG, and EREG in colorectal tumors (Fig. 6N–S). Similarly, ERK phosphorylation was elevated in xenograft tumors derived from OSBPL2 KD CRC cells (Fig. 6T). These results demonstrate that OSBPL2 defect facilitates colorectal growth by activating ERK signaling.

### The function of OSBPL2 on metastasis is mediated by PARP1 through ZEB1

**Fig. 7:**
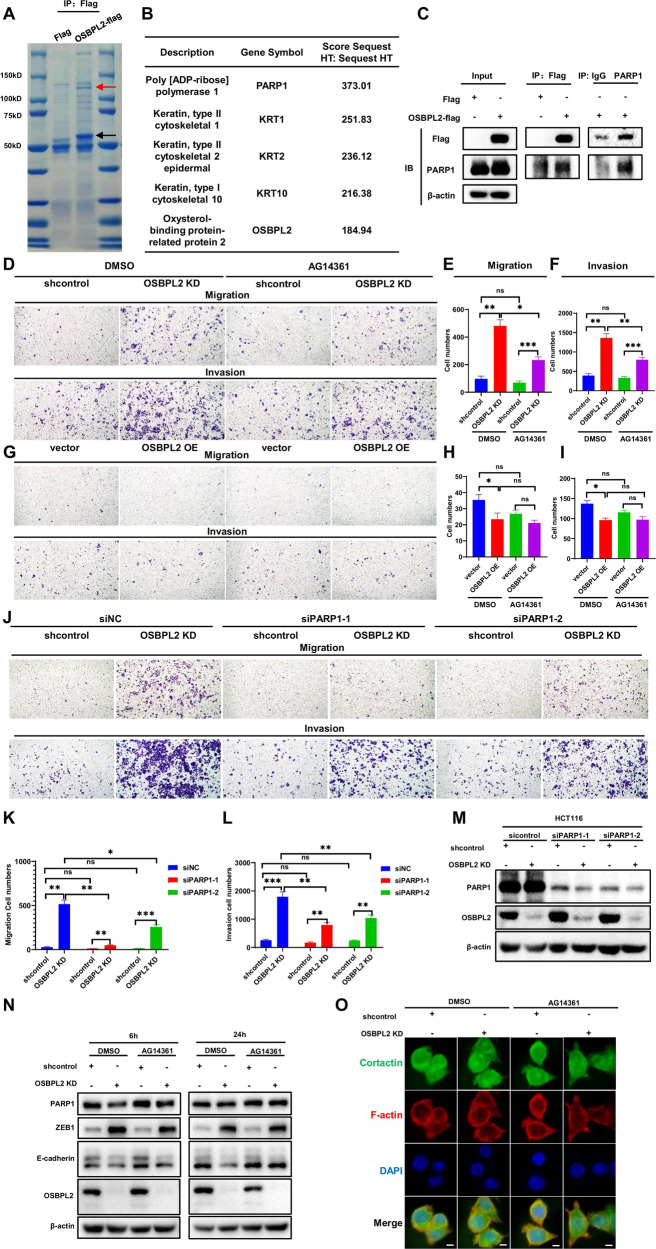
The function of OSBPL2 on metastasis is dependent on PARP1 through ZEB1. **A** The co-IP experiment of determining the proteins interacted with OSBPL2 (depicted in red arrows). **B** Identifying the proteins in (**A**) by mass spectrum. **C** The co-IP assay showing the interaction between OSBPL2 and PARP1. **D**–**F** Transwell assays were performed to assess the migration and invasion abilities of shcontrol and OSBPL2 KD HCT116 cells with treatment of DMSO and AG14361. **G**–**I** Transwell assays were carried out to evaluate the migration and invasion ability of vector and OSBPL2 OE HCT116 cells with treatment of DMSO and AG14361. **J**–**L** The shcontrol and OSBPL2 KD HCT116 cells were transient transfected with siNC, siPARP1-1 and siPARP1-2, then be analyzed by transwell assay. NC negative control. **M** The shcontrol and OSBPL2 KD HCT116 cells were transient transfected two PARP1 siRNAs, the expressions of PARP1, OSBPL2 were detected by western blot, sicontrol was used as control. **N** The shcontrol and OSBPL2 KD HCT116 were treated with AG14361 at concentrations of 5 μM for 6 h or 24 h, PARP1, OSBPL2, ZEB1, E-cadherin were detected by western blot, DMSO was used as control. β-actin was served as loading control. **O** The shcontrol and OSBPL2 KD HCT116 were treated with AG14361 at concentrations of 1 μM, respectively. Immunofluorescence was carried out to detect invadopodia with stimulation of Collagen I for 24 h. Cortactin were labelled in green, F-actin was labelled in red, and the cell nucleus were stained by DAPI (in blue). Scale bars, 10 μm.

A co-immunoprecipitation (co-IP) assay and mass spectrometry were used to identify the proteins interacting with OSBPL2. An interacting protein band appeared between 100 and 150 kDa (Fig. 7A), and PARP1 was identified based on the highest score (Fig. 7B). The interaction between OSBPL2 and PARP1 was verified using a co-IP assay (Figs. 7C and S6A). PARP1 plays a multifaceted role in DNA repair, chromatin remodeling, and PARylation (poly [ADP] ribosylation) of various EMT-related proteins such as E-cadherin, Snail1, ZEB1, Twist1, p65, and SMADs [41–43]. We constructed a PARP1 expression vector and siRNA and applied the PARP1 specific inhibitor AG14361 to validate the relationship between OSBPL2 and PARP1. The IC50 concentration of AG14361 was 13.33 μM, which suppressed cell proliferation without inducing cell death (Fig. S6B).

AG14361 blocked the capabilities of migration and invasion induced by OSBPL2 in HCT116 cells (Fig. 7D–I). CRC cells were more sensitive to the PARP1 inhibitor, whereas the level of OSBPL2 was low. Hence, OSBPL2 competes with this inhibitor by trapping PARP1 during CRC metastasis. Knockdown of PARP1 by the two siRNAs markedly inhibited the metastatic function of OSBPL2 (Fig. 7J–M). However, the PARP1 overexpression did not impede the metastatic function of OSBPL2 (Fig. S6C–F). Otherwise, PARP1 had no impact on CRC cell adhesion to Collagen I or on the activation of ERK signaling (Fig. S6G–I).

Moreover, AG14361 blocked the upregulation of ZEB1 earlier and the downregulation of E-cadherin expression late (Fig. 7N). It substantially blocked invadopodia formation induced by OSBPL2 knockdown, which may be related to the phosphorylation of cortactin (Figs. 7O and S6J). These data indicate that PARP1 mediates OSBPL2 function in EMT through ZEB1.

### OSBPL2 protects against colorectal tumor metastasis following tail vein and intrasplenic injection

**Fig. 8:**
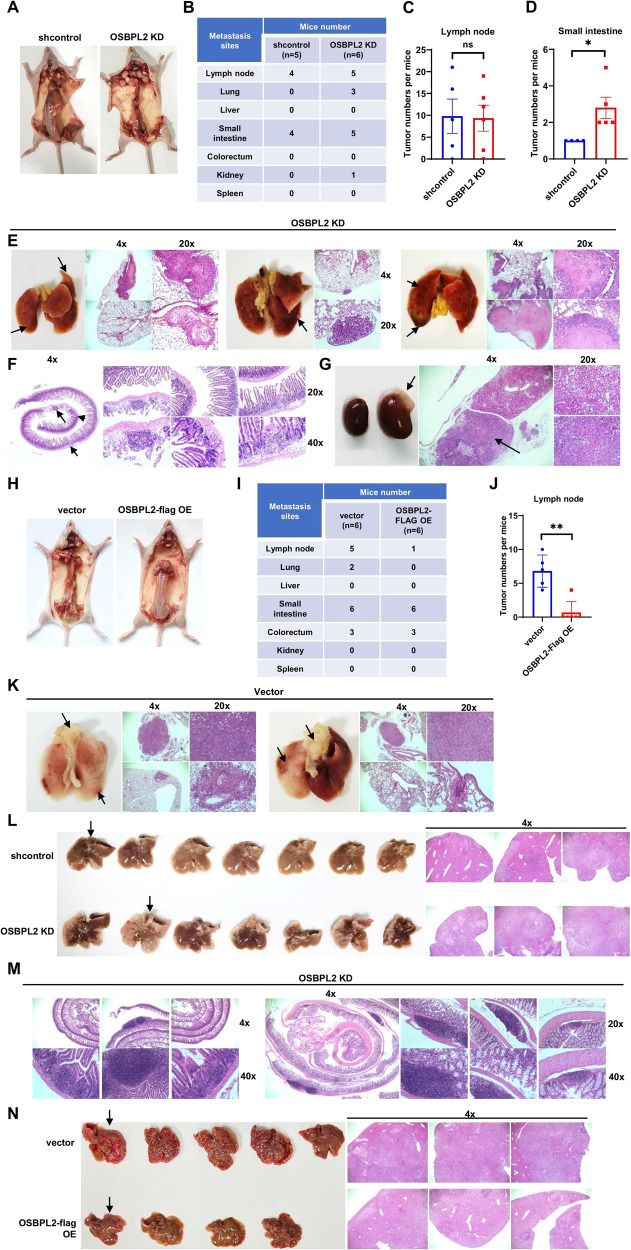
OSBPL2 protects against colorectal tumor metastasis *via* tail vein injection and intrasplenic injection. **A**–**D** Mice were dissected after being injected with shcontrol and OSBPL2 KD HT29 (*n* = 11) cells via the tail vein. Representative anatomical images (**A**), statistics of tumor metastatic sites (**B**), differences in lymph node metastasis (**C**), and differences in small intestine metastasis (**D**). Representative images and H&E-stained scans of the lungs (**E**), small intestine (**F**), and kidneys (**G**) in OSBPL2 KD HT29 group. **H**–**J** Mice were dissected after being injected with vector and OSBPL2-flag OE HT29 (*n* = 12) cells via the tail vein. The weight of the mice was measured, and their condition was observed every 3-4 days. Representative anatomical images (**H**), statistics of tumor metastatic sites (**I**), and differences in lymph node metastasis (**J**). **K** Representative images and H&E-stained scans of the lungs in vector HT29 group. The metastatic nodules were marked in black arrows. **L** After establishing a liver metastasis model of intrasplenic injection with shcontrol and OSBPL2 KD HT29 (*n* = 14) cells, the mice were dissected. The images of livers were shown. Representative H&E-stained images were scanned for the liver tissues indicated by the black arrows. **M** Representative H&E-stained images of the small intestine (left) and colorectum (right) of mice injected with OSBPL2 KD HT29 cells. **N** After establishing a liver metastasis model of intrasplenic injection with vector and OSBPL2 OE HCT116 (*n* = 9) cells, the mice were dissected. The weight of the mice was measured, and their condition was observed every 3–4 days. The images of livers were shown. Representative H&E-stained images were scanned for the liver tissues indicated by the black arrows.

To evaluate the function of OSBPL2 in CRC metastasis in vivo, we used tail vein and intrasplenic injection models. The tail vein injection model showed that the lack of OSBPL2 resulted in more metastatic nodules in the lungs, small intestines, and kidneys (Figs. 8A–D and S7A–F). Although there were no differences in the number of metastatic nodules in the lymph nodes, they were larger in the OSBPL2 KD group (Figs. 8C and S7B). Likewise, colorectal length was slightly shorter in the OSBPL2 KD group (Fig. S7F). The histological morphologies of the metastatic nodules are shown in Fig. 8E–G. Overexpression of OSBPL2 restrained tumor metastasis to the lymph nodes and lungs (Figs. 8H–K and S7G–L).

The intrasplenic injection model showed that OSBPL2 depletion increased the liver metastasis burden (Fig. 8L, S8A–S8C). As shown in Figs. 8M, and S8D, E, mice injected with OSBPL2 KD HT29 cells had more metastatic nodules in the small intestines and larger metastatic nodules in the colorectum. In contrast, OSBPL2 overexpression eliminated liver metastasis tumors (Figs. 8N and S8F–I). These results indicate that OSBPL2 protects against CRC metastasis.

### SCH772984 and AG14361 relieve colorectal tumor progression induced by OSBPL2 defect

**Fig. 9:**
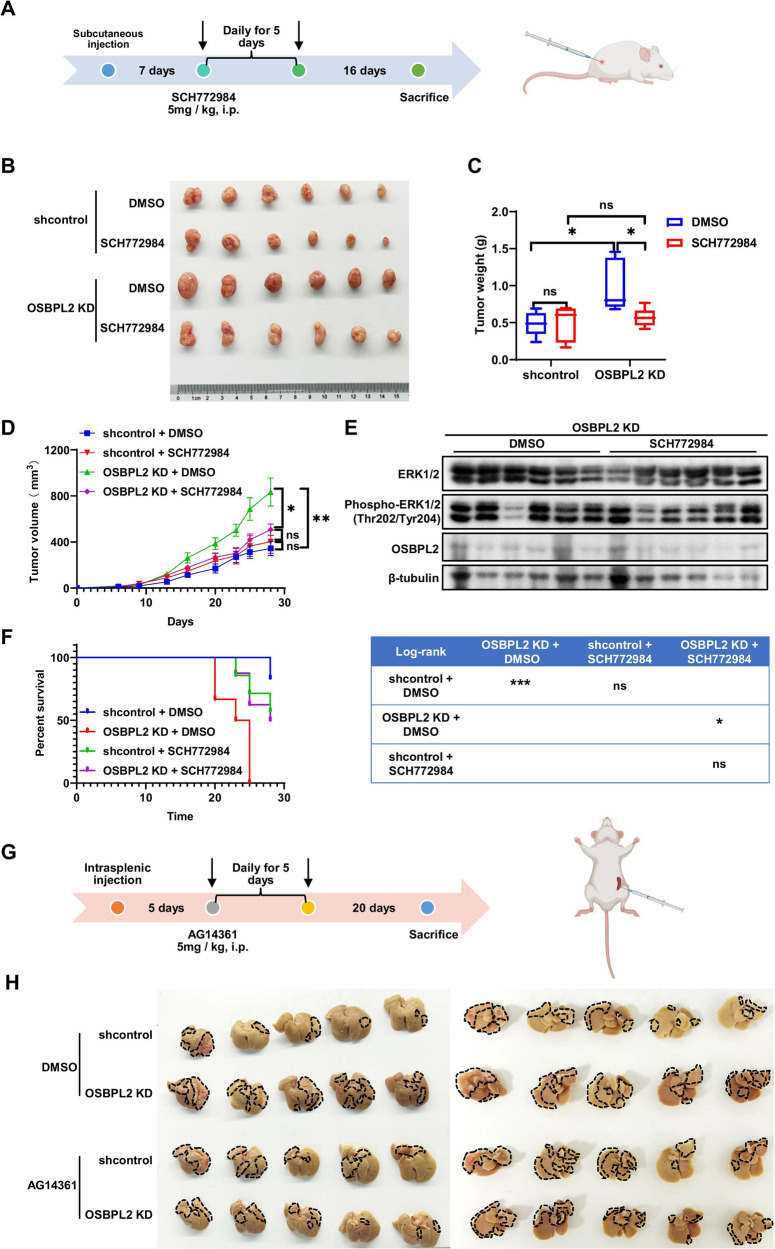
SCH772984 and AG14362 inhibits OSBPL2 defect induced CRC progression. **A** The flow chart of xenograft tumor model derived from shcontrol and OSBPL2 KD HCT116 cells with treatment of SCH772984. i.p: intraperitoneal injection. Images (**B**) and weights (**C**) of the tumors harvested from nude mice and tumor growth curve (**D**) of shcontrol and OSBPL2 KD HCT116 cells with treatment of SCH772984 respectively, DMSO as control. **E** The protein levels of total ERK, Phospho-Erk1/2 (Thr202/Tyr204) and OSBPL2 in xenograft tumors(B) were detected by western blot, β-tubulin was utilized as the loading control. **F** Kaplan–Meier analysis of mouse survival and statistical analyses in **A** are depicted. **G** The flow chart of tumor metastasis model derived from shcontrol and OSBPL2 KD HCT116 cells with treatment of AG14361. **H** The images of livers in (**F**) were shown. The areas of liver tumors were marked in black dotted line.

As ERK signaling and PARP1 play important roles OSBPL2 function, SCH772984 and AG14361 were used to treat CRC characterized by OSBPL2 deficiency. The effect of SCH772984 on CRC growth was evaluated using a xenograft tumor model (Fig. 9A). The ERK inhibitor SCH772984 significantly alleviated colorectal tumor growth induced by OSBPL2 knockdown, with reduced average tumor weights and volumes by 41.19% and 39.24%, respectively (Fig. 9B–D). SCH772984 treatment markedly suppressed the activation of ERK signaling in tumors derived from OSBPL2 KD CRC cells (Fig. 9E). Furthermore, SCH772984 substantially improved the poor survival induced by OSBPL2 knockdown (Fig. 9F). The effect of AG14361, a PARP1 inhibitor, on CRC metastasis was examined using a liver metastasis model (Fig. 9G). AG14361 markedly reduced the number of liver metastatic nodules induced by the loss of OSBPL2 (Fig. 9H). These data suggest that SCH772984 and AG14361 suppressed CRC progression in vivo.

## Discussion

**Fig. 10:**
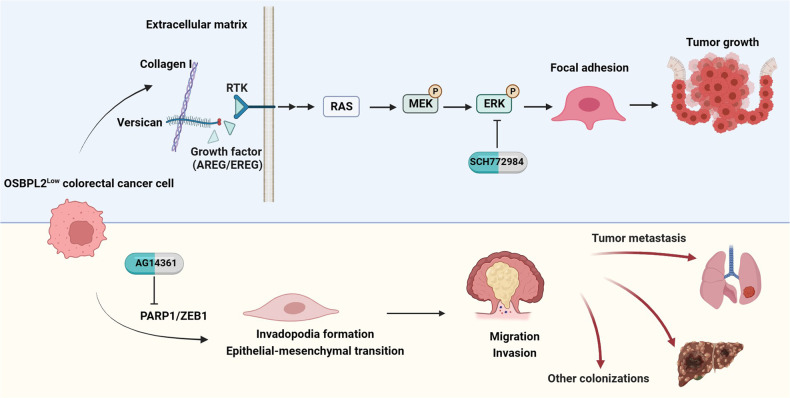
Schematic diagram of OSBPL2 function on CRC. OSBPL2 defect promoted Collagen I-induced CRC cell growth, focal adhesion, migration and invasion. Independently, OSBPL2 regulated ERK signaling *via* VCAN/AREG/EREG axis in CRC cell growth and modified EMT through PARP1/ZEB1. The ERK inhibitor SCH772984 and PARP1 inhibitor AG14361 alleviated CRC progression induced by loss of OSBPL2.

ORPs modulate oncogenic signaling as oncogenes or tumor suppressors in testicular, hematologic, pancreatic, lung, and liver cancers [44–47]. Here, we show that OSBPL2 (ORP2) predicts a good prognosis for metastatic CRC. OSBPL2 defect promoted Collagen I-induced CRC cell growth, focal adhesion, migration, and invasion but impeded CRC cell proliferation without ECM. OSBPL2 regulated ERK signaling *via* the VCAN/AREG/EREG axis in CRC cell growth and modified EMT through PARP1. Most importantly, OSBPL2 inhibited colorectal tumor growth and distant metastasis, which were blocked by SCH772984 and AG14361 (Fig. 10).

We discovered that OSBPL2 alleviated Collagen I-induced focal adhesion by impairing FAK autophosphorylation at Y397, which binds to SRC family kinases. FAK regulates the expression of the cell cycle protein cyclin D or apoptosis through SRC/ERK, JNK, or PI3K/AKT signaling, thereby affecting cell survival and tumor growth [48]. FAK is highly expressed in metastatic CRC and is modulated by lipids [49, 50]. Although OSBPL2 accelerated CRC cell proliferation without the ECM, the reduction of focal adhesion and FAK signaling led to a decrease in stable OSBPL2 OE cell-derived colorectal tumor growth in the Collagen I-rich TME. Additionally, focal adhesion remodeling of the actin cytoskeleton is linked to anoikis and invasion [51]. Hence, OSBPL2 attenuated Collagen I-induced focal adhesion, thereby reducing invadopodia formation. Targeting both OSBPL2 and FAK may be an effective combination therapy for CRC. However, further investigation is required to validate this hypothesis [52].

In the present study, the VCAN/AREG/EREG axis synergistically regulated ERK signaling induced by decreased OSBPL2 expression. The expressions of AREG and EREG are higher in left-sided CRC than in right-sided CRC and is more sensitive to anti-EGFR therapy [53, 54]. In this study, we only used the ERK inhibitor SCH772984 to suppress the growth of tumors derived from OSBPL2^Low^ CRC cells. However, combined inhibition of the ECM genes, VCAN, and ERK signaling may be more effective for preventing OSBPL2 loss induced colorectal tumor growth.

Finally, we elucidated the correlation between OSBPL2 and PARP1 expression in CRC metastases. PARP1 is an ADP-ribosyltransferase enzyme that binds to single- and double-stranded DNA breaks during the DNA damage response [55]. We found that PARP1 is necessary for the metastatic function of OSBPL2. The expression and mutations of PARP1 must be examined before improving OSBPL2 level for CRC therapy, except in the early stages of CRC, in which PARP1 is overexpressed [56]. PARP1 expression is visualized using radiolabeled PARP1 inhibitors in clinical settings [57]. The mechanisms of action of its inhibitors, such as nicotinamide adenine dinucleotide (NAD) analogs, involve synthetic lethality and PARP1 trapping. However, dual-target inhibitors of PARP1 have been developed to overcome tumor toxicity and resistance [58]. As shown in Figs. 7 and 9, OSBPL2 deficiency can ameliorate the sensitivity of CRC to PARP1 inhibitors, and the PARP1 inhibitor AG14361 could eliminate metastasis induced by OSBPL2 defect.

This study had some limitations. As other ECM molecules, such as fibronectin and laminin, may coordinate with Versican to allow OSBPL2-regulated ERK signaling during CRC cell growth. Decrease of OSBPL2 stimulated growth of CRC cells, slightly induced VCAN accumulation and activated ERK signaling (Fig. S5D–F), the molecular mechanism needs to be further explored. Correspondingly, it is noteworthy how OSBPL2 regulates ECM degradation by matrix metalloproteinase and plasminogen activator systems.

Furthermore, based on IHC image of OSBPL2 in CRC, OSBPL2 was transported extracellularly. The future research direction of OSBPL2 as a secretory protein may be associated with exosomes acting on colorectal tumor microenvironment. And the diagnosis and drugs of OSBPL2 in CRC will be urgently developed.

Briefly, this study illustrates that OSBPL2 defect collaborates with ECM Collagen I to promote colorectal tumor growth and metastasis through two independent pathways and provides the basis for appropriate therapy using ERK and PARP1 inhibitors to treat OSBPL2^Low^ CRC.

## Materials and methods

### Reagents and equipment

The details of reagents and equipment used in this study were shown in Table S3.

### Cell culture

We established cell culture systems for five cell lines using DMEM or RPMI 1640 media supplemented with 10% FBS and 1% P/S. Specifically, HEK293T cells and CRC cell lines HCT116 and SW620 were cultured in DMEM, while the other two CRC cell lines, HT29 and LoVo, were cultured in RPMI 1640 medium. All cells are cultured in a humidified incubator at a constant temperature of 37 °C with 5% CO_2_. All cells were sourced from the Shanghai Life Academy of Sciences cell library (Shanghai, China).

### Tissue microarrays

From January 2015 to December 2018, we collected 627 tissue samples (adjacent normal tissues, *n* = 312; CRC tissues, *n* = 315), from Yangpu Hospital, Tongji University (Shanghai, China) with the consent of surgical patients. CRC patients at stages I to IV provided all samples used in this study, which were reviewed by experienced pathologists prior to being utilized. A TMA was created using formalin-fixed and paraffin-embedded specimens. The utilization of all tissue samples has obtained approval from the Hospital Ethics Committee. We downloaded pan-cancer samples containing mRNA sequencing data of 19,131 samples from the UCSC database (https://xenabrowser.net/). We conducted a log_2_(x + 0.001) transformation on the OSBPL2 mRNA expression levels of 34 cancer samples and generated a bar chart illustrating the expression of OSBPL2 in pan-cancer.

### Immunohistochemistry (IHC) and H&E staining

The tissue slides featuring paraffin sections were subjected to deparaffinization with xylene and ethanol. The activity of endogenous peroxidase was blocked by subjecting the sample to a 15-min treatment with 3% H_2_O_2_ at room temperature. To perform antigen retrieval on tissue slides, they were heated in a citrate buffer for 20 min, followed by blocking with a DPBS solution containing 10% FBS at 37 °C for 15 min. Subsequently, OSBPL2 or Ki67 was added to the tissue slides and incubated overnight at 4°C. The staining was performed with the DAB substrate, followed by incubating the slides with a biotin-conjugated secondary antibody using a general SP kit. We designed a two-dimensional scoring system to assess the immunoreactivity level of the prepared slides. The scoring ranges from 0 to 3 for staining intensity and from 0 to 4 for the proportion of positive cells. During the process of H&E staining of tissue slides, the first step involves fixing the slides. Subsequently, the slides are stained by adding Harris-modified hematoxylin solution and 1% aqueous eosin Y solution, and the staining process is continued for 1–2 min until completion. Dehydration was carried out by immersing the slides in ascending alcohol solutions and xylene. The data obtained from the experiment were analyzed using the Image J software.

### OSBPL2 knockdown and overexpression

The pCDH-PURO vector was used to clone full-length OSBPL2. Subsequently, the construction of short hairpin RNA (shRNA) for OSBPL2 was achieved using the pLKO.1-PURO vector, with use of the following target sequences:

shOSBPL2-1: 5′-GGATTACTTTGAGCGGAATTT-3′;

shOSBPL2-2: 5′-GGGAGAAACGTATGAATTAAT-3′;

shOSBPL2-3: 5′-GAAGATTTAGGATTCAGATTT-3′;

scramble control: 5′-CCTAAGGTTAAGTCGCCCTCG-3′.

Lipofectamine 3000 was utilized to facilitate co-transfection of packaging plasmids (psPAX2/pMD2.G) and viral vectors (pCDH and pLKO.1) in HEK293T cells. Following a 48-h incubation period, the cell medium was passed through a 0.45 μm filter. Following a 48-h viral medium infection, cells were subjected to selection using puromycin to establish stable cell lines.

### RNA isolation and real-time PCR

We extracted total RNA from the target cells using Trizol reagent, following the standard protocol. After determining the RNA concentration, the total RNA samples were reverse transcribed using RT master mix. Real-time PCR was performed using SYBR Green. All aforementioned steps were conducted in compliance with the provided protocols. Data were evaluated using the 2^−ΔΔCt^ method, with real-time PCR primer details available in Supplementary Table S2.

### Western blot

For total protein extraction, the target cells were first washed with PBS, followed by cell lysis using RIPA lysis buffer containing protease and phosphatase inhibitors. After BCA quantification, the samples were mixed with loading buffer and boiled for 10 min to obtain total protein samples. Protein separation was carried out using SDS-PAGE, and the total proteins were subsequently transferred onto PVDF membranes. After blocking with 5% non-fat milk for 1 h, the membranes were incubated overnight at 4 °C with primary antibodies. The antibodies used in this study are as follows: OSBPL2, DDDDK-Tag, E-Cadherin, PARP1, Vimentin, FAK, Phospho-FAK-Y397, Phospho-FAK-Y576/577, Phospho-FAK-Y925, Versican, ERK1/2, Phospho-ERK1/2 (Thr202/Tyr204), ZEB1, β-actin and β-tubulin. Visualization of the proteins was achieved through ECL methodology following incubation with secondary antibodies targeting Goat anti-Mouse IgG and Goat anti-Rabbit IgG. The protein levels of OSBPL2 and β-actin were quantified using Image J software.

### Cell proliferation detection

In this study, we used a CCK8 kit to determine the viability of target cells. An appropriate amount of CCK8 reagent was added to the 96-well plate to be tested. The plate was then incubated in a cell culture incubator at 37 °C for 2 h. After incubation, we measured the absorbance at 450 nm using a microplate reader to assess cellular viability. Additionally, we performed colony formation assays to assess cell proliferation capability. We seeded 200 CRC cells into each individual well of a 6-well plate, cultured them for approximately 10 days, and subsequently harvested and fixed the cells for staining. For the cloning formation experiment that required Collagen I (40 μg/ml) or fibronectin (40 μg/ml) stimulation, Collagen I or fibronectin was added to DPBS and thoroughly mixed on ice. Subsequently, the mixture was added to the plate, sealed with parafilm, and then incubated overnight at 4 °C. After removing excess liquid, cells were seeded as described previously. For the cloning formation experiment involving laminin stimulation (1–2 μg/cm^2^), laminin was added to DPBS and thoroughly mixed on ice. The resulting mixture was then applied to the plate, sealed with parafilm, and incubated for 2 h at 37 °C. After being washed three times with DPBS, the plate was ready for cell seeding. When visible cell colonies were observed, the cells were harvested. Cell fixation was performed using 4% paraformaldehyde at 37 °C for 15 min. After being washed three times with PBS, the cells were stained with a crystal violet staining solution. For cells subjected to flow cytometry analysis, after harvesting, they are first fixed with paraformaldehyde, and then permeabilized using 0.2% Triton X-100. Subsequently, the prepared Ki67 primary antibody is added to the cells and incubated on ice for 1 h. Cell proliferation analysis was carried out with the aid of BD Accuri™ C6 flow cytometer. Analysis of the results was performed with BD Accuri™ C6 software.

### Cell adhesion assay

The CRC cells were subjected to a process of washing and starving for 8 h. Following the aforementioned procedures, place the pre-coated 96-well plate with collagen I at room temperature for 1 h. After aspirating the liquid from the wells, add serum-free culture medium (0.1% BSA) to the plate. Then, seed the cells (5 × 10^4^) into the plate and incubate for 2 h. Cells were incubated for another 4 h in complete medium. Finally, a 2-h incubation with CCK8 reagent was conducted before detection.

### Migration and invasion quantification

For the detection of cell migration and invasion capabilities, we used Transwell chambers. The target cells were digested from the dishes, centrifuged, and washed with serum-free culture medium. Subsequently, the cells were resuspended in an appropriate volume of serum-free culture medium and then seeded into the upper chamber. For the wells of the 24-well plate, medium containing 10% FBS was added beforehand. The chamber was coated with matrigel for invasion assay. After a 48-h incubation, stain the fixed CRC cells with crystal violet, followed by their detection and quantification through utilization of an inverted optical microscope and ImageJ software, respectively.

### Immunofluorescence staining

Fix the CRC cells or tissues on the slides with 4% paraformaldehyde for 15 min, followed by washing with PBS three times, and then permeabilize using 0.2% Triton X-100. Incubate the prepared slides with primary antibodies against Paxillin, Cortactin, E-cadherin, TRITC Phalloidin and DDDDK-Tag. The secondary antibodya were FITC Goat Anti-Mouse IgG, FITC Goat Anti-Rabbit IgG, Cy3 Goat Anti-Rabbit IgG and Cy3 Goat Anti-Mouse IgG. Olympus BX53 microscope was employed for fluorescence confocal image capturing. Later, Image J software was used for analyzing the average fluorescence intensity.

### VCAN and PARP1 RNA interference

Human PARP1 small interfering RNAs (siRNAs) were procured from TSINGKE (Beijing, China). The manufacturer’s protocol was followed for siRNA transfection, utilizing Lipofectamine RNAiMAX. Provided below are the siRNA target sequences:

siVCAN-1: 5′-GATTGGTCAGGACTACAAA-3′;

siVCAN-2: 5′-CACCTGTTATCCUACTGAA-3′;

siVCAN-3: 5′-GGCTGTTATGGAGATAAGA-3′;

siPARP1-1: 5′-GGTTGACAGAGATTCTGAA-3′;

siPARP1-2: 5′-GAAAGTGTGTTCAACTAAT-3′.

### Co-IP

Using the method mentioned in the Western blot section, the CRC cells were lysed, and the total protein extracted was used for Co-IP assays. IgG and protein A/G agarose beads were used to pre-clear the protein. The pre-clearing process was conducted for 1 h at 4 °C. Following the pre-clearing process, fresh protein A/G magnetic beads and a specific antibody (or IgG) were added to the lysates. Finally, incubate the mixture overnight at 4 °C. Sequential washing with PBS was done three times on the beads the following day. Elution of the washed beads was carried out using protein sample loading buffer, followed by SDS-PAGE.

### Coomassie Brilliant Blue-staining of protein gels

The gels were treated with Coomassie Brilliant Blue on a rocking platform for 30 min and destained using a solution of Milli-Q water.

### Animal experiments and treatments

We purchased six-week-old male nude mice (BALB/cA-nu/nu) from Hangzhou Ziyuan Experimental Animal Technology Co., Ltd. These mice were housed in appropriate living conditions, with a 12-h light–dark cycle. We provided the mice with adequate food and sterile drinking water, and we regularly changed them. Additionally, we cleaned the cages and replaced the bedding on a regular basis. Animals are randomized based on the body weight. To initiate the subcutaneous tumor model, 65 mice were injected with stable CRC cell lines (1 × 10^6^–3 × 10^6^ cells) subcutaneously in the flank. Tumor volume (V) was assessed one week after injection by measuring with digital calipers at intervals of 3–4 days, with this formula: V (mm^3^) = 0.5 × length × width^2^. Tumors were harvested and weighed before their tumor volume reached 1000 mm³. For SCH772984 treatment, CRC cells were injected following the aforementioned protocol. When tumor volumes reached 30 mm^3^, these mice (n = 20) were intraperitoneal injected with DMSO or SCH772984 group (5 mg/kg) daily for 5 days. The subsequent measurements of tumor volume and tumor harvesting were executed as previously delineated. The endpoint for survival was a tumor exceeding 500 mm^3^.

For the tumor metastasis model, 2 × 10^6^ CRC cells were evenly mixed, then injected into the tail vein of mice (*n* = 23). The weights of the mice were measured, and their conditions was observed every 3-4 days. These mice were euthanized 35 ± 2 days after surgery. For the hepatic metastasis model, a solution of 2–4 × 10^6^ HCT116 or HT29 cells was injected into the splenic parenchyma after opening the upper abdominal wall. We observed the condition of mice (*n* = 23) and measured their body weight every 3–4 days. These mice were euthanized 42 ± 2 days after surgery. For AG14361 treatment, 5 days after the intrasplenic injection, the mice (*n* = 20) were intraperitoneal injected with DMSO or AG14361 group (5 mg/kg) daily for 5 days. The use of animals for this study adhered to the guidelines set by the Animal Center of Tongji University.

### Statistical analysis

For statistical analyses in the study, GraphPad Prism was utilized. The data presented in this study is primarily shown as mean ± SEM (standard error of the mean). For immunohistochemistry results, we display the data as mean ± SD (standard deviation). For TCGA dataset, expression between tumor and normal samples were evaluated via employment of R software (version 3.6.4), with significant analysis executed by means of non-paired Wilcoxon Rank Sum and Signed Rank Tests. For comparisons between two groups, we employed paired or unpaired Student’s t-tests. For multiple group comparisons, multiple t-tests were performed, with one t-test per row. The overall survival was generated using the Kaplan-Meier method and analyzed with the log-rank test. In this study, each experiment was followed by at least 3 replicates, *P* < 0.05 indicates a statistically significant difference between the data.

### Supplementary information

Supplementary file

Original Data File

Reproducibility Checklist

## Data Availability

The RNA-seq data generated during the current study are available in NCBI Sequence Read Archive (SRA), BioProject ID: PRJNA1003685, https://www.ncbi.nlm.nih.gov/sra/PRJNA1003685. All data analyzed in this study are available from the corresponding author on reasonable request.
