# Supplementary material for: Collagen I-induced VCAN/ERK signaling and PARP1/ZEB1-mediated metastasis facilitate OSBPL2 defect to promote colorectal cancer progression
Source: Cell Death Dis. 2024 Jan 24;15(1):85. doi: 10.1038/s41419-024-06468-1 (PMC10808547; doi:10.1038/s41419-024-06468-1)
Supplement: Supplementary file 1 — Supplementary file [file 41419_2024_6468_MOESM1_ESM.docx]

Supplementary Materials for

**Collagen Ⅰ-induced VCAN/ERK signaling and PARP1/ZEB1-mediated metastasis facilitate OSBPL2 defect to promote colorectal cancer progression**

Kang Lin^1,2^ *et al.*

*Corresponding author. Email: lmbin@hotmail.com; heluwei88@126.com

**This PDF file includes:**

Supplementary Text

Figs. S1 to S8

Tables S1 to S3


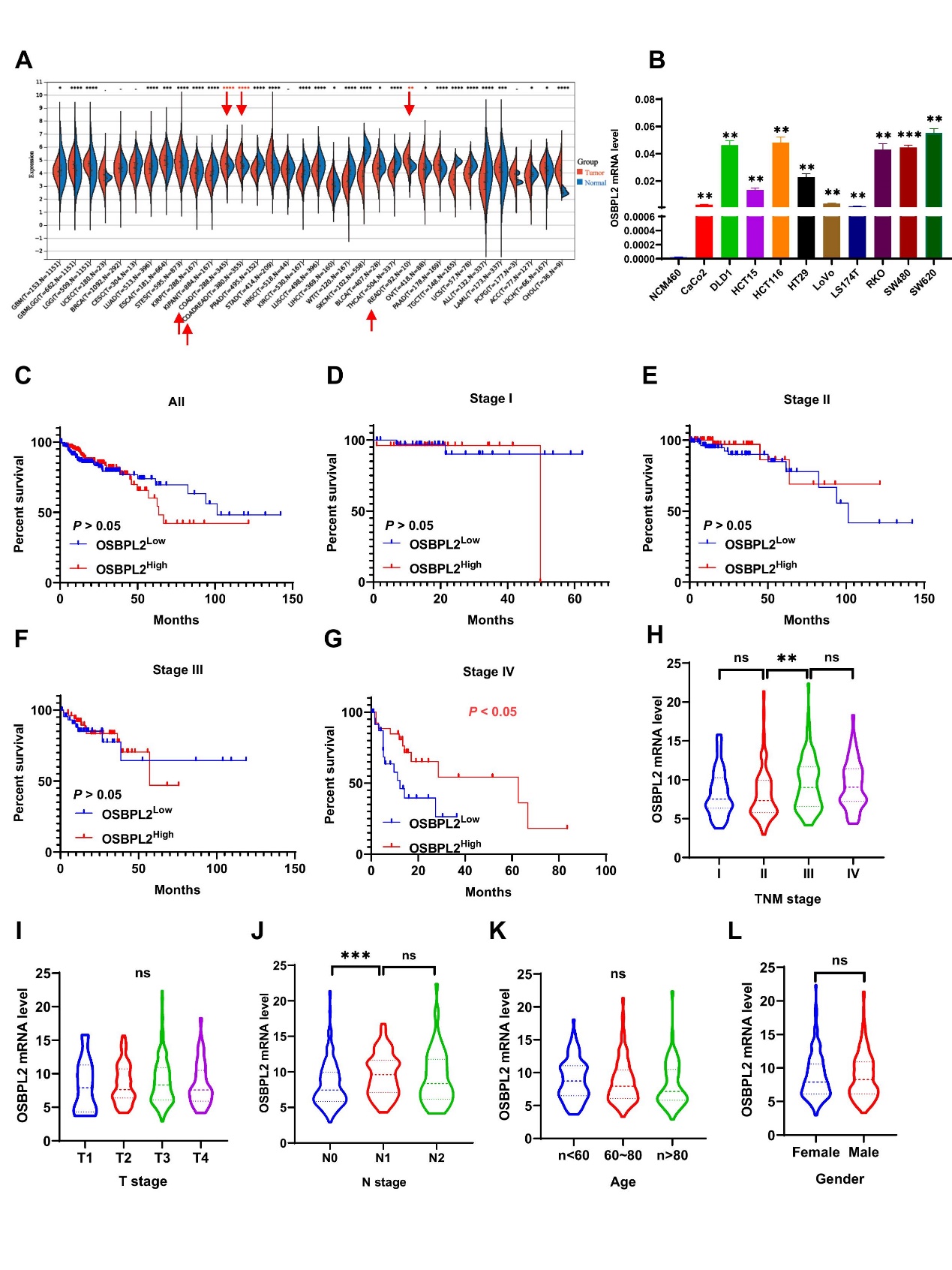


**Figure S1 Low OSBPL2 expression is associated with poor prognosis in Stage Ⅳ CRC patients (from TCGA database)**

A. The mRNA expression of OSBPL2 in tumor and normal tissues (from TCGA pan-cancer samples database), COAD (colon adenocarcinoma) and READ (rectal adenocarcinoma) were marked in red arrows. B. Real-time PCR was used to detect the expression difference of OSBPL2 in normal colorectal cells (NCM460) and colorectal cancer cells (CaCo2, DLD1, HCT15, HCT116, HT29, LoVo, LS174T, RKO, SW480, and SW620). C-G. Tumor samples in colorectal cancer were divided into high and low expression groups based on the median OSBPL2 expression level, and the survival of the two groups was analyzed using the Kaplan-Meier method in all stages (C), stage I (D), stage II (E), stage III (F), and stage IV (G). H. The mRNA expression of OSBPL2 in different TNM stages of colorectal cancer. I-L. The mRNA expression of OSBPL2 in different T stages (I), N stages (J), ages (K), and genders (L) of colorectal cancer. Mean ± SD. *, P < 0.05; **, P < 0.01; ***, P < 0.001; ns, no significance.


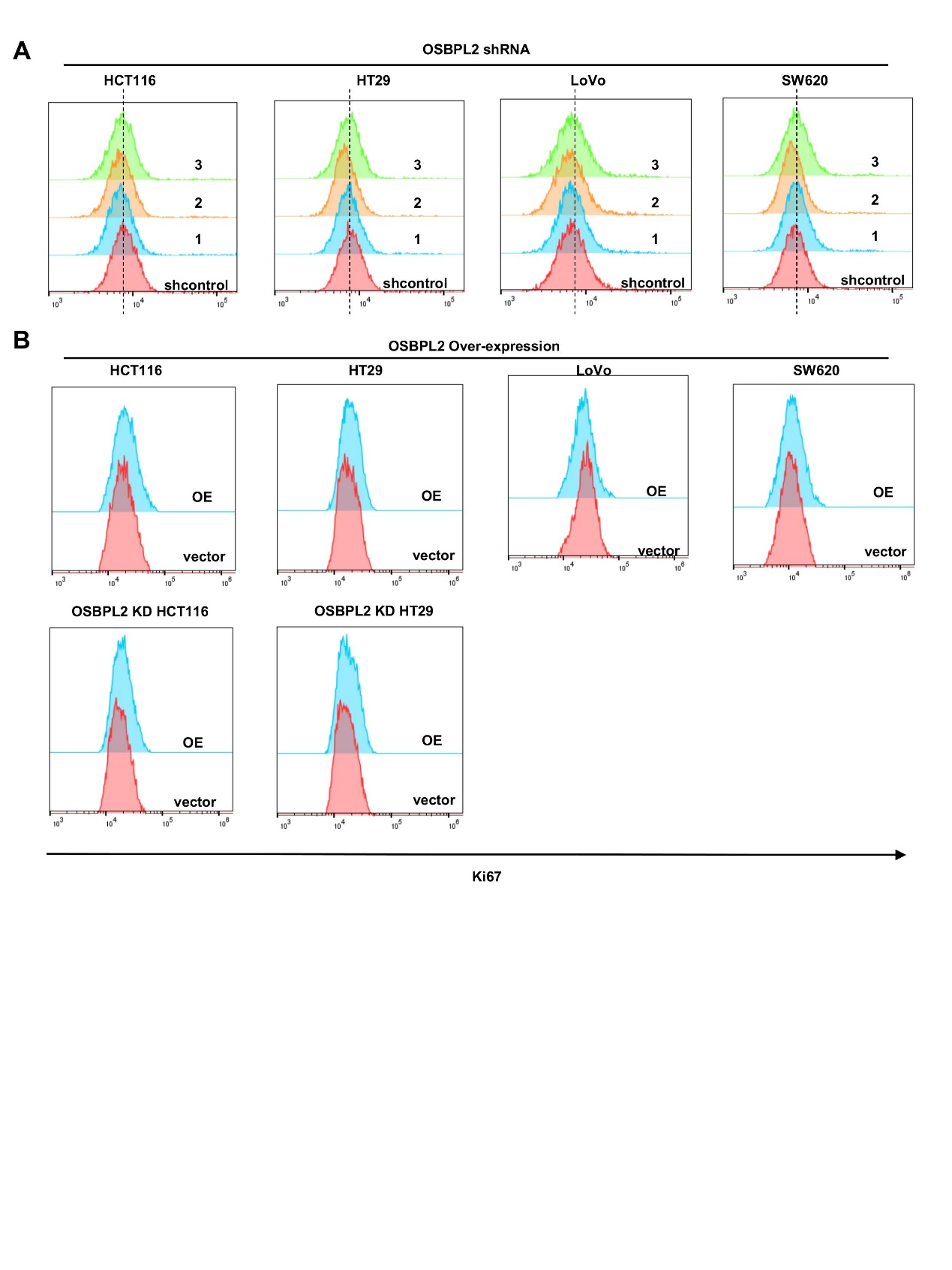


**Figure S2 Ki67 staining of Figure 2**

A. Flow cytometry was used to analyze ki67 intensity in HCT116, HT29, LoVo, and SW620 cells after knockdown of OSBPL2, shcontrol was served as control. B. After overexpression of OSBPL2, Ki67 intensity was analyzed in HCT116, HT29, LoVo, SW620, OSBPL2 KD HCT116 and HT29 cells by flow cytometry, vector was served as control.

**
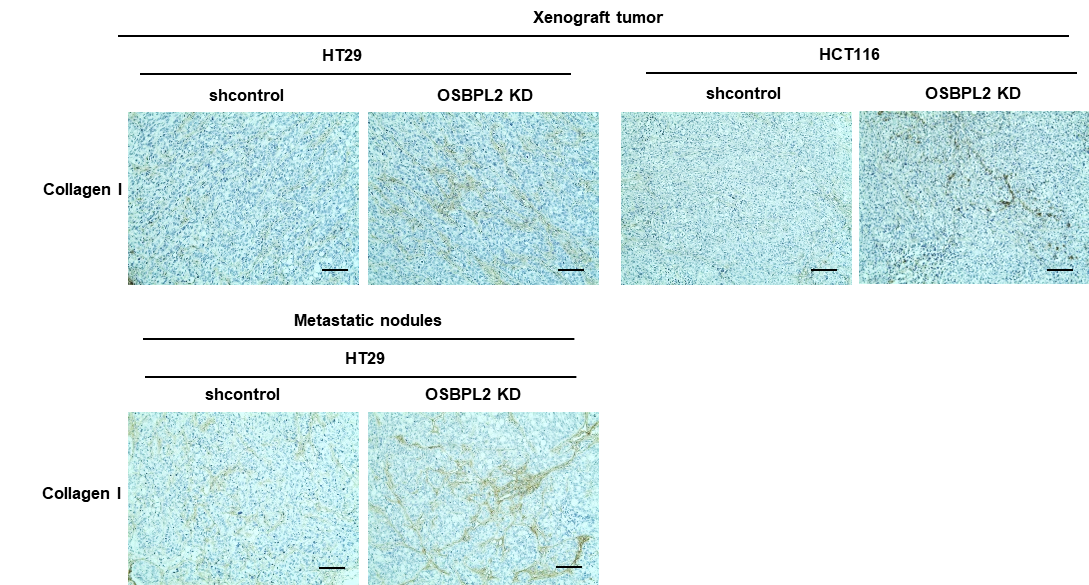
**

**Figure S3 Collagen I staining of Figure 3N-3O**

IHC analysis of Collagen I expression of Figure 6A, 8A. Scale bars, 20 μm.

**
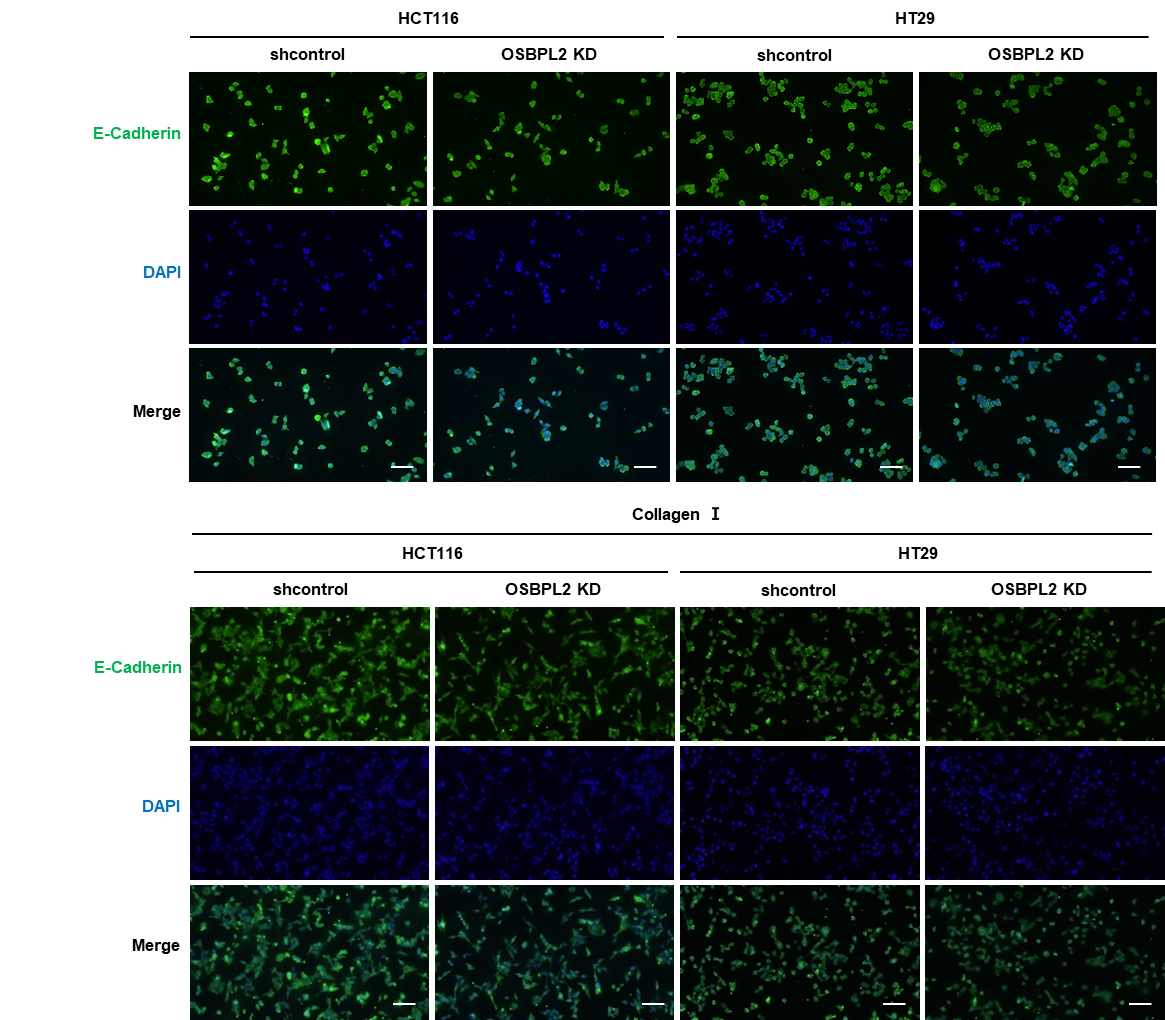
**

**Figure S4 E-cadherin staining of Figure 4H-4I**

Immunofluorescence staining was employed to analyze the intensity of E-cadherin in shcontrol and OSBPL2 KD HCT116 and HT29 cells with or without Collagen I. E-cadherin was labelled in green, cell nucleus was stained by DAPI (in blue), scale bar 20μm.

**
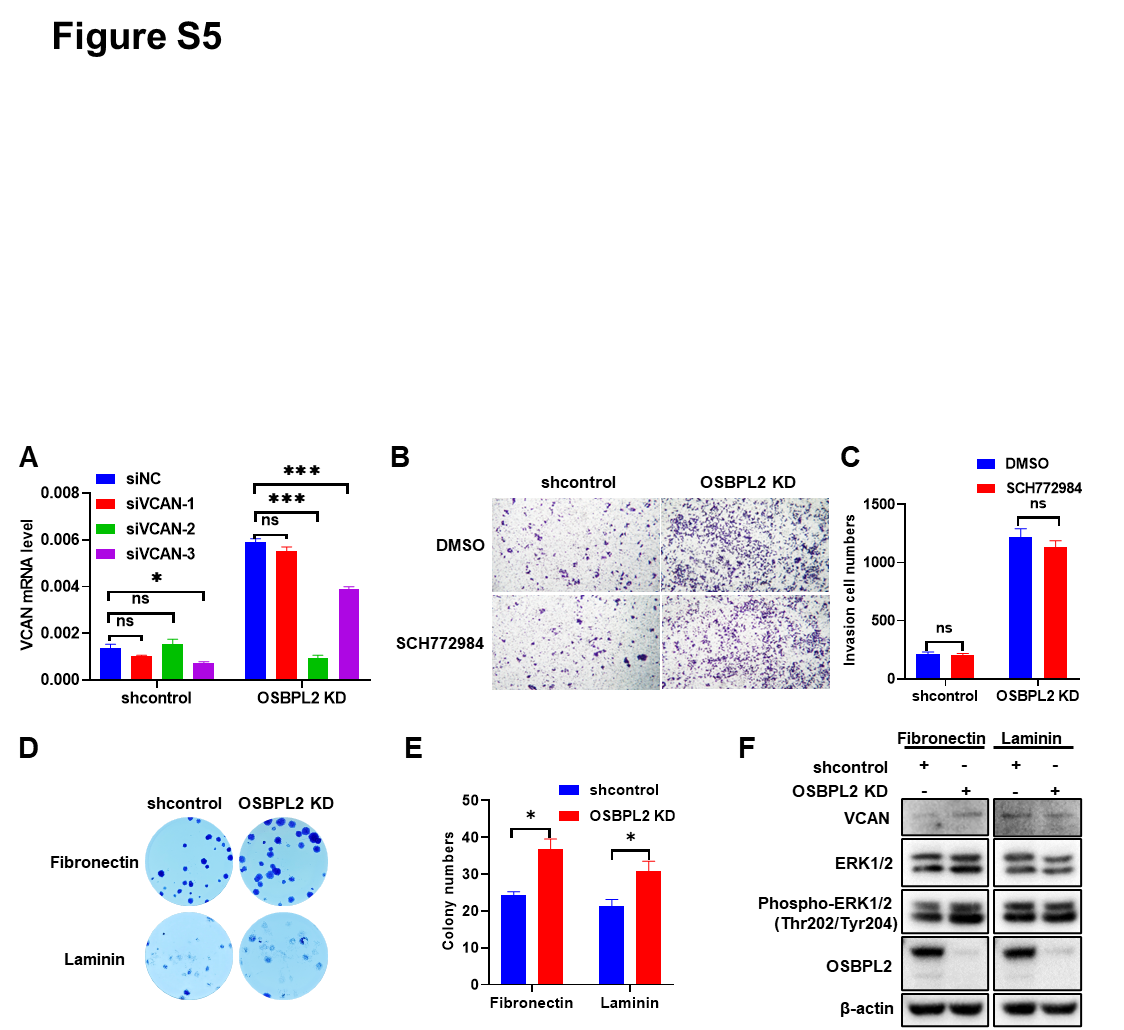
**

**Figure S5 Supplementary materials of Figure 5**

A. After knockdown Versican by three VCAN siRNAs, the expressions of VCAN were examined by real-time PCR, ACTB was used as a housekeeping gene. B-C. The shcontrol and OSBPL2 KD HCT116 were treated with SCH772984 (2.5nM), respectively, then be detected by transwell invasion assay. D-E. Colony formation assays were performed on OSBPL2 KD stable HCT116 cell lines with the incubation of fibronectin or laminin, shcontrol was used as a control. F. Western blotting was employed to detect the protein levels of VCAN, ERK1/2, Phospho-ERK1/2 (Thr202/Tyr204) and OSBPL2 in shcontrol and OSBPL2 KD HCT116 cells. The treatment groups were stimulated with Fibronectin, and Laminin, respectively. β-actin was utilized as the loading control.


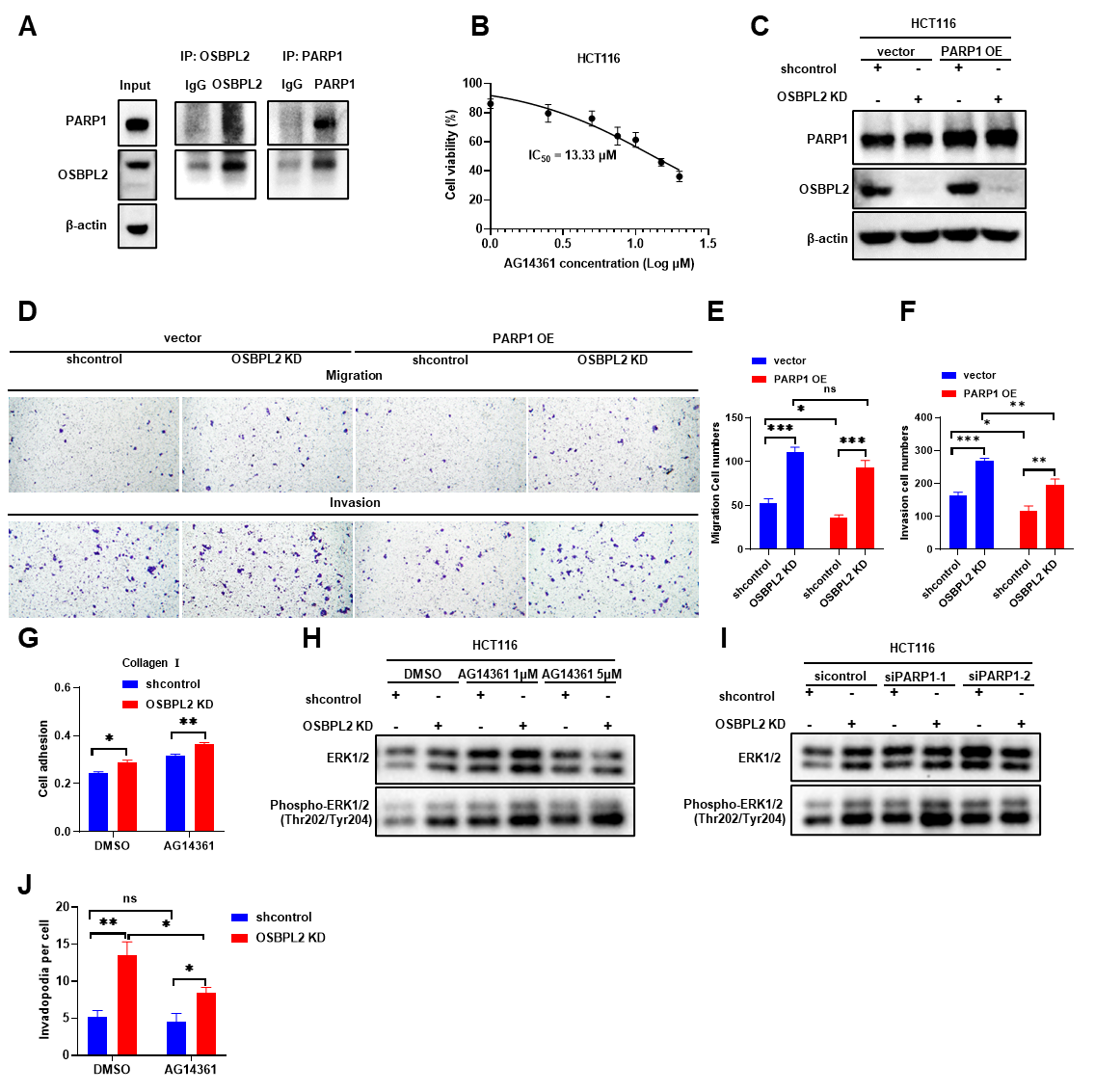


**Figure S6 Supplementary materials of Figure 7**

A. The co-IP assay showing the interaction between OSBPL2 and PARP1 at endogenous level. B. The IC_50_ of AG14361 in HCT116 cells. Cell viabilities of HCT116 cells treated with AG14361 at 1, 2.5, 5, 7.5, 10, 15, 20 μmol/L. DMSO was used as control. The IC_50_ (half maximal inhibitory concentration) was analyzed by Graphpad software. C. The shcontrol and OSBPL2 KD HCT116 cells were transient transfected with PARP1 cDNA, the expressions of PARP1 and OSBPL2 were detected by western blotting, vector were used as control. β-actin was served as loading control. D-F. The shcontrol and OSBPL2 KD HCT116 cells were transient transfected with vector and PARP1, then be analyzed by transwell assay. G. The Collagen I-mediated cell adhesion assay was performed on the shcontrol and OSBPL2 KD HT29 cells, followed by treatment with AG14361 at concentrations of 5uM. H-I. The protein levels of total ERK and Phospho-ERK1/2 (Thr202/Tyr204) were measured by western blot, followed by treatment with AG14361 or PARP1 siRNAs. J. Statistic of Figure 7O.


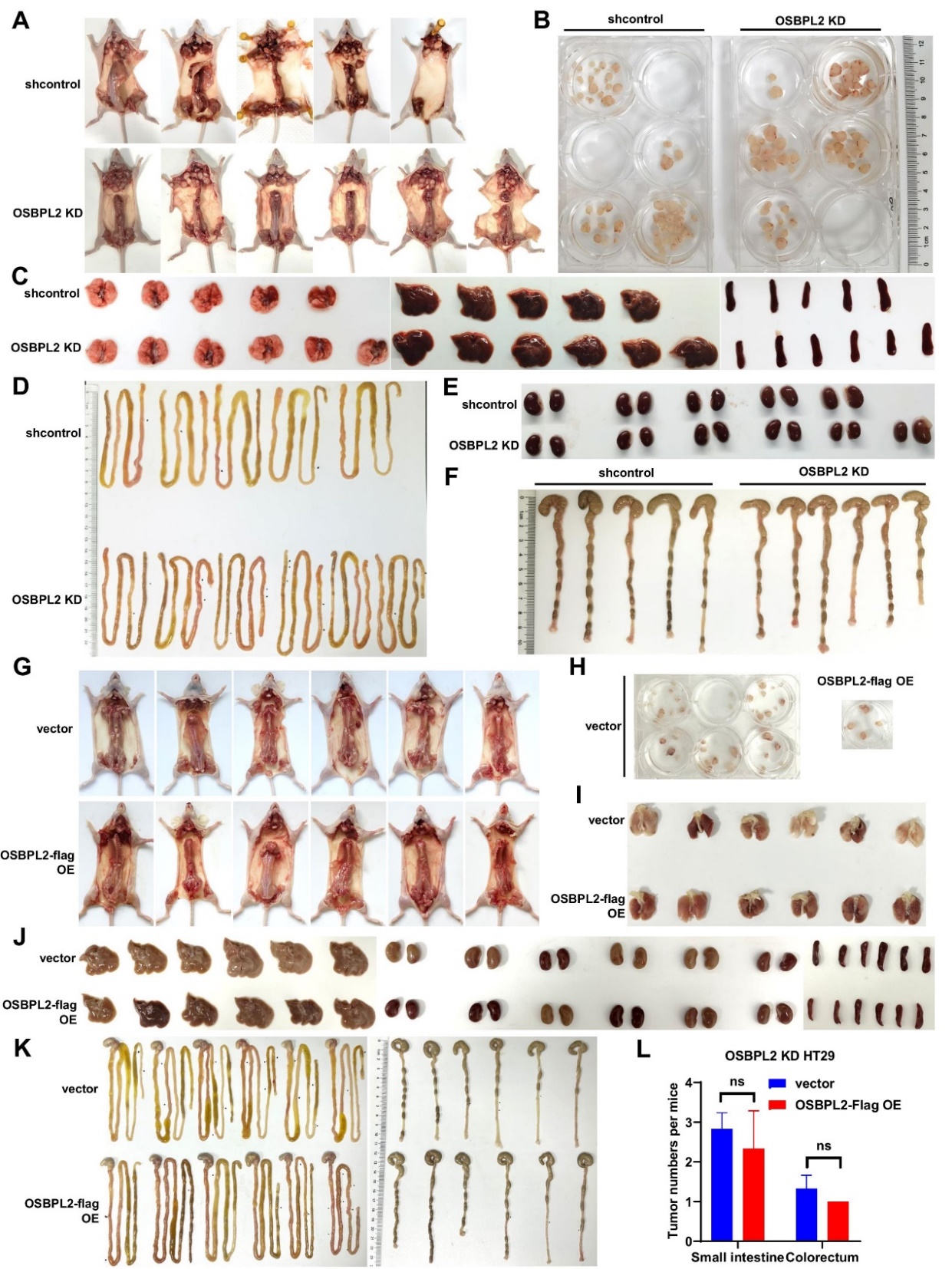


**Figure S7 The experimental details in Figure 8A-8K.**

A-F. The ventral view of dissected mice in Figure 8A (A), lymph node metastases in a 6-well plate (B), the dissected lung (left), liver (middle), and spleen (right) of mice (C), the dissected small intestine (D), kidney (E), and colorectum (F). G-L. The ventral view of dissected mice in Figure 8G (G), the lymph node metastases in a 6-well plate (H), the dissected lung (I), liver (left), kidney (middle), and spleen (right) of mice (J), the dissected small intestine (left) and colorectum (right) (K). L. The colorectal length was measured and metastatic nodules of small intestine and colorectum were marked in black dots.


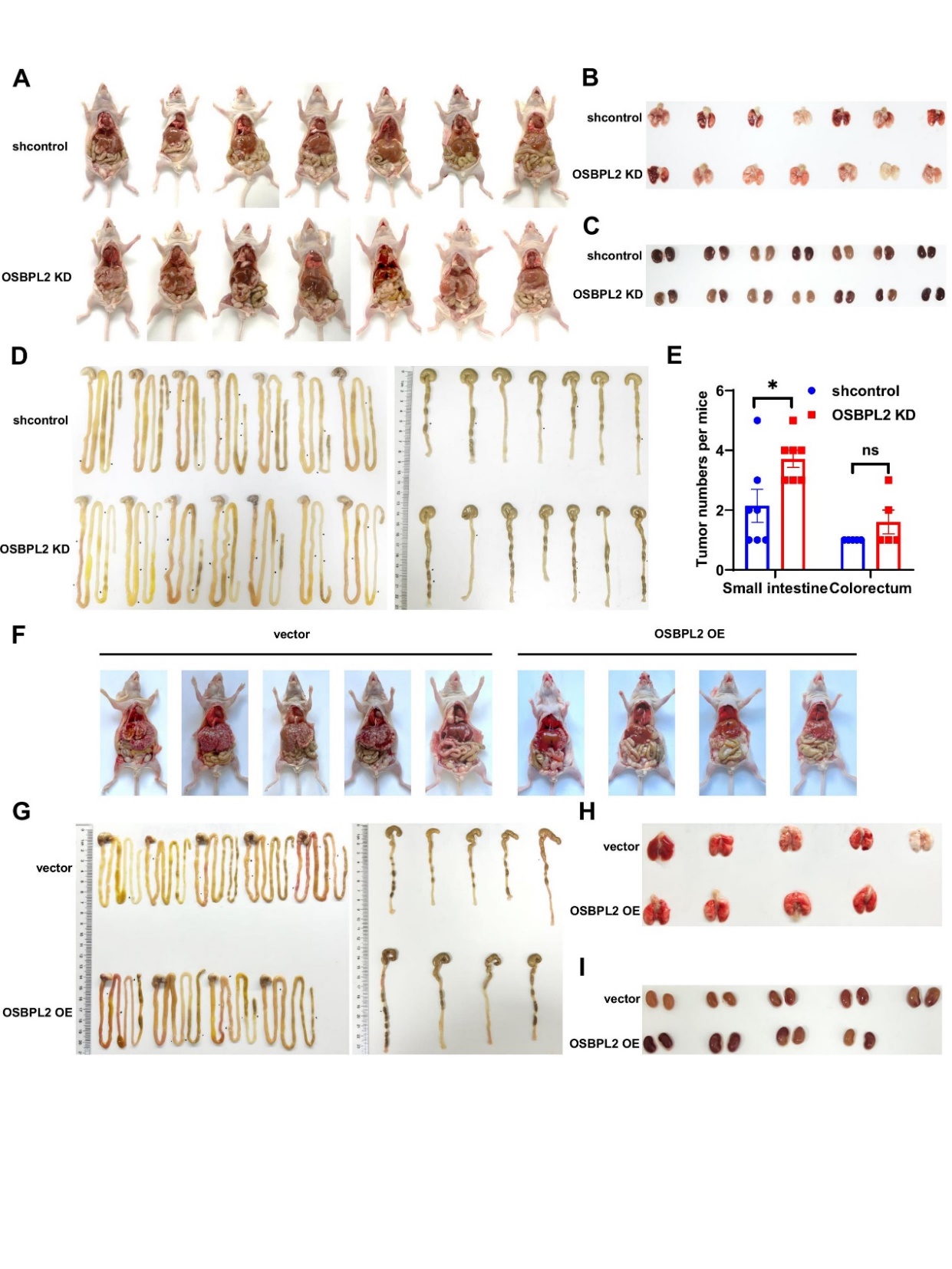


**Figure S8 The experimental details in Figure 8L-8N.**

A-E. The ventral view of dissected mice in Figure 8L (A), the dissected lung (B), kidney (C), small intestine and colorectum (D), the numbers of metastatic tumors in the small intestine and colorectum (E). F-I. The ventral view of dissected mice in Figure 8N (F), the dissected small intestine (left) and colorectum (right) (G), lung (H), and kidney (I). The metastatic nodules of small intestine and colorectum were marked in black dots.

**Table S1 The list of differential genes (OSBPL2 KD vs shcontrol)**

| **Gene name** | **Fold change**  **(OSBPL2 KD vs shcontrol)** | ***P* value** | **KEGG pathway** |
| --- | --- | --- | --- |
| AREG | 3.46 | 2.15E-46 | MAPK signaling  ErbB signaling  PI3K-Akt signaling  Hippo signaling  Colorectal cancer |
| VCAN | 2.96 | 5.83E-38 | Cell adhesion molecules |
| SEMA3A | 3.68 | 1.59E-37 |  |
| KRT7 | 0.17 | 4.16E-37 |  |
| DKK1 | 0.46 | 1.36E-27 |  |
| NAV3 | 3.78 | 3.86E-27 |  |
| CYP24A1 | 2.01 | 1.34E-24 |  |
| FOXC1 | 2.18 | 5.43E-20 |  |
| NT5E | 2.26 | 1.99E-19 |  |
| SLC16A6 | 3.17 | 1.92E-18 |  |
| ARL4C | 0.52 | 5.09E-16 |  |
| EREG | 2.20 | 8.05E-16 | MAPK signaling  ErbB signaling  PI3K-Akt signaling  Colorectal cancer |
| ALDH1A3 | 0.56 | 9.87E-16 |  |
| TACSTD2 | 0.16 | 3.30E-15 |  |
| OSBPL2 | 0.44 | 3.70E-15 |  |

**Table S2 The real-time PCR primers**

| **Gene** | **Forward primers (5’-3’)** | **Reverse primers (5’-3’)** |
| --- | --- | --- |
| OSBPL2 | AGAGGTGACCACCTGAGAAAGG | GTTGATCCTCCAGAGCAGCTTG |
| CDH1 | GCCTCCTGAAAAGAGAGTGGAAG | TGGCAGTGTCTCTCCAAATCCG |
| VIM | AGGCAAAGCAGGAGTCCACTGA | ATCTGGCGTTCCAGGGACTCAT |
| SNAIL1 | TGCCCTCAAGATGCACATCCGA | GGGACAGGAGAAGGGCTTCTC |
| SNAIL2 | ATCTGCGGCAAGGCGTTTTCCA | GAGCCCTCAGATTTGACCTGTC |
| TWIST1 | GCCAGGTACATCGACTTCCTCT | TCCATCCTCCAGACCGAGAAGG |
| TWIST2 | GCAAGATCCAGACGCTCAAGCT | ACACGGAGAAGGCGTAGCTGAG |
| ZEB1 | GGCATACACCTACTCAACTACGG | TGGGCGGTGTAGAATCAGAGTC |
| ZEB2 | AATGCACAGAGTGTGGCAAGGC | CTGCTGATGTGCGAACTGTAGG |
| CTNNB1 | CACAAGCAGAGTGCTGAAGGTG | GATTCCTGAGAGTCCAAAGACAG |
| AREG | GCACCTGGAAGCAGTAACATGC | GGCAGCTATGGCTGCTAATGCA |
| EREG | CTTATCACAGTCGTCGGTTCCAC | GCCATTCAGACTTGCGGCAACT |
| VCAN | TTGGACCTCAGGCGCTTTCTAC | GGATGACCAATTACACTCAAATCAC |
| ACTB | CACCATTGGCAATGAGCGGTTC | AGGTCTTTGCGGATGTCCACGT |

**Table S3 The details of reagents and equipment used in this study**

| **Full name** | **Abbreviation** | **Catalogue** | **Company** | **Address** |
| --- | --- | --- | --- | --- |
| Fetal bovine serum | FBS | F8318 | Sigma | St. Louis, MO, USA |
| Penicillin/streptomycin | P/S | 15140163 | Gibco | Carlsbad, CA, USA |
| Dulbecco's Modified Eagle Medium | DMEM | 11995073 | Gibco | Carlsbad, CA, USA |
| RPMI 1640 medium |  | 11875119 | Gibco | Carlsbad, CA, USA |
| DPBS |  | 14190250 | Gibco | Carlsbad, CA, USA |
| SP kit |  | SP-9000 | ZSGB-BIO | Beijing, China |
| DAB substrate |  | SP-9000 | ZSGB-BIO | Beijing, China |
| Harris modified Hematoxylin solution |  | HHS16 | Sigma | St. Louis, MO, USA |
| Eosin Y solution |  | HT110216 | Sigma | St. Louis, MO, USA |
| Xylene |  | 214736 | Sigma | St. Louis, MO, USA |
| pCDH-PURO vector |  | 46970 | Addgene | Watertown, MA, USA |
| pLKO.1-PURO vector |  | 8453 | Addgene | Watertown, MA, USA |
| Lipofectamine 3000 |  | L3000015 | Invitrogen | Carlsbad, CA, USA |
| psPAX2 |  | 12259 | Addgene | Watertown, MA, USA |
| pMD2.G |  | 12260 | Addgene | Watertown, MA, USA |
| 0.45 μm filter |  | PN4614 | PALL | Show Low, AZ, USA |
| Puromycin |  | ant-pr-1 | Invivogene | San Diego, USA |
| Trizol reagent |  | 15596026 | Invitrogen | Carlsbad, CA, USA |
| RT master mix |  | RR036B | Takara | Mountain View, CA, USA |
| SYBR Green |  | Q511-03 | Vazyme | Nanjing, China |
| PBS |  | ST476 | Beyotime | Shanghai, China |
| RIPA buffer |  | P0013B | Beyotime | Shanghai, China |
| Protease and phosphatase inhibitor cocktail |  | P1045 | Beyotime | Shanghai, China |
| SDS-PAGE |  | PG111 | Epizyme | Shanghai, China |
| Polyvinylidene difluoride | PVDF | ISEQ00010 | Millipore | Billerica, MA, USA |
| OSBPL2 |  | 14751-1-AP | Proteintech | Rosemont, IL, USA |
| DDDDK-Tag |  | AE005 | Abclonal | Wuhan, China |
| E-Cadherin |  | 20874-1-AP | Proteintech | Rosemont, IL, USA |
| PARP1 |  | 13371-1-AP | Proteintech | Rosemont, IL, USA |
| Vimentin |  | 10366-1-AP | Proteintech | Rosemont, IL, USA |
| FAK |  | A21578 | Abclonal | Wuhan, China |
| Phospho-FAK-Y397 |  | AP030 | Abclonal | Wuhan, China |
| Phospho-FAK-Y576/577 |  | AP0536 | Abclonal | Wuhan, China |
| Phospho-FAK-Y925 |  | AP1098 | Abclonal | Wuhan, China |
| Versican |  | A19655 | Abclonal | Wuhan, China |
| ERK1/2 |  | 9102 | Cell Signaling | MA, USA |
| Phospho-ERK1/2 (Thr202/Tyr204) |  | 9101 | Cell Signaling | MA, USA |
| ZEB1 |  | 21544-1-AP | Proteintech | Rosemont, IL, USA |
| β-actin |  | 66009-1-Ig | Proteintech | Rosemont, IL, USA |
| β-tubulin |  | 66240-1-lg | Proteintech | Rosemont, IL, USA |
| Goat anti-Mouse IgG |  | AS003 | Abclonal | Wuhan, China |
| Goat anti-Rabbit IgG |  | AS014 | Abclonal | Wuhan, China |
| CCK8 solution |  | C0043 | Beyotime | Shanghai, China |
| Collagen I |  | 40125ES10 | Yeasen | Shanghai, China |
| Fibronectin |  | 40105ES08 | Yeasen | Shanghai, China |
| Laminin |  | 114956-81-9 | Sigma | St. Louis, MO, USA |
| Paraformaldehyde |  | P0099 | Beyotime | Shanghai, China |
| Crystal Violet Staining Solution |  | C0121 | Beyotime | Shanghai, China |
| Transwell chambers |  | 3422 | Corning | Kenneburg, ME, USA |
| KI67 |  | 27309-1-AP | Proteintech | Rosemont, IL, USA |
| Matrigel |  | 40183ES08 | Yeasen | Shanghai, China |
| Paxillin |  | 10029-1-Ig | Proteintech | Rosemont, IL, USA |
| Cortactin |  | 11381-1-AP | Proteintech | Rosemont, IL, USA |
| TRITC Phalloidin |  | 40734ES75 | Yeasen | Shanghai, China |
| FITC Goat Anti-Mouse IgG |  | AS001 | Abclonal | Wuhan, China |
| FITC Goat Anti-Rabbit IgG |  | AS011 | Abclonal | Wuhan, China |
| Cy3 Goat Anti-Rabbit IgG |  | AS007 | Abclonal | Wuhan, China |
| Cy3 Goat Anti-Mouse IgG |  | AS008 | Abclonal | Wuhan, China |
| Lipofectamine RNAiMAX |  | 13778150 | Invitrogen | Carlsbad, CA, USA |
| IgG and protein A/G agarose beads |  | Cal#sc-2003 | Santa Cruz Biotechnology | CA, USA |
| loading buffer |  | LT103 | Epizyme | Shanghai, China |
| Coomassie Brilliant Blue |  | P0017F | Beyotime | Shanghai, China |
| SCH772984 |  | HY-50846 | MedChemExpress | New Jersey, USA |
| AG14361 |  | HY-12032 | MedChemExpress | New Jersey, USA |
| GraphPad Prism software |  |  | GraphPad | San Diego, CA, USA |
| Flow cytometer |  |  | BD Biosciences | Franklin Lakes, NJ, USA |
