# Supplementary material for: Collagen I-induced VCAN/ERK signaling and PARP1/ZEB1-mediated metastasis facilitate OSBPL2 defect to promote colorectal cancer progression
Source: Cell Death Dis. 2024 Jan 24;15(1):85. doi: 10.1038/s41419-024-06468-1 (PMC10808547; doi:10.1038/s41419-024-06468-1)
Supplement: Supplementary file 2 — Original Data File [file 41419_2024_6468_MOESM2_ESM.docx]

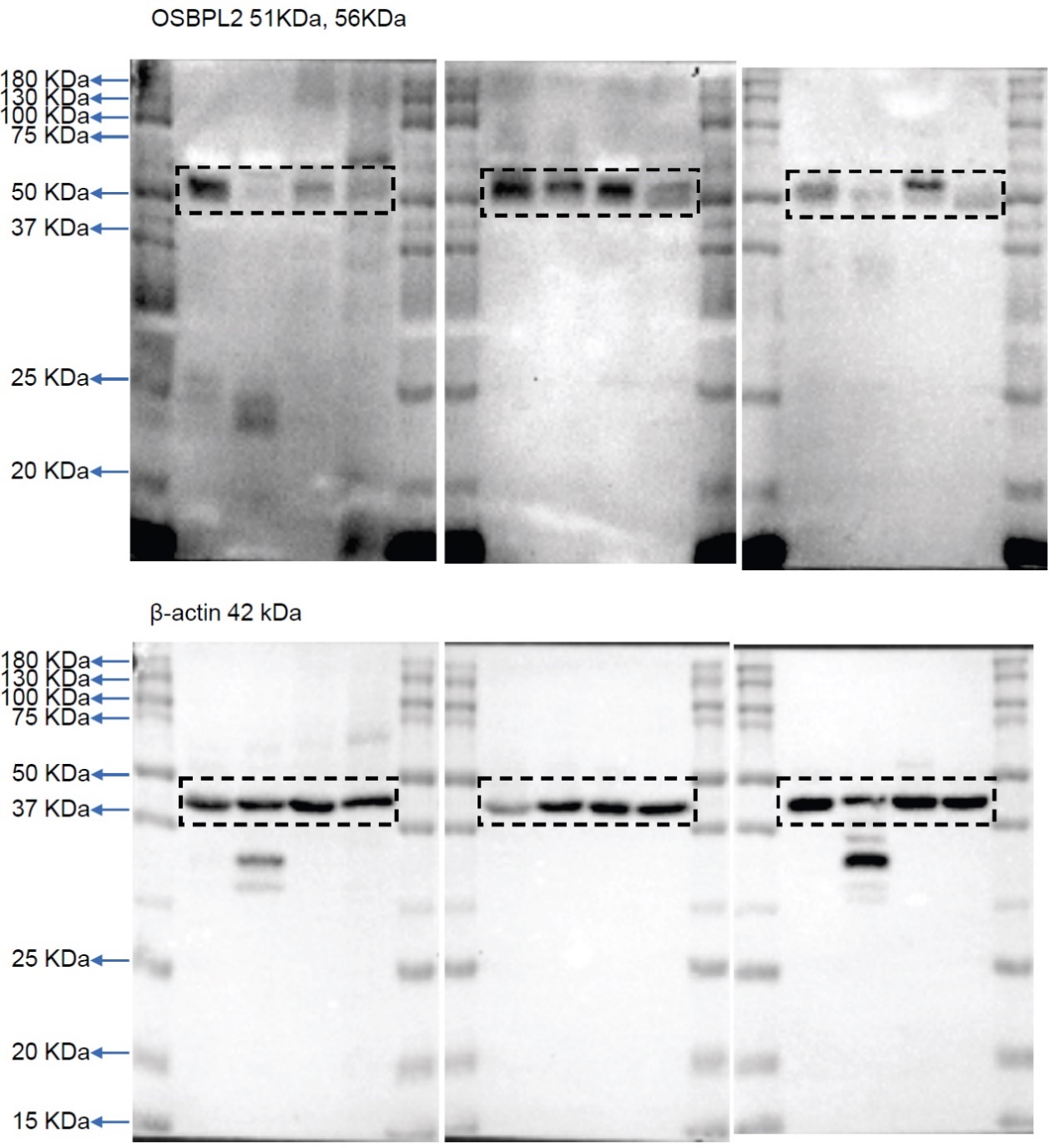


**Supplemental Figure S1** *related to Figure 1*

Immunoblots related to Figure 1, immunoblots of OSBPL2 and β-actin obtained from colorectum and liver tissue.


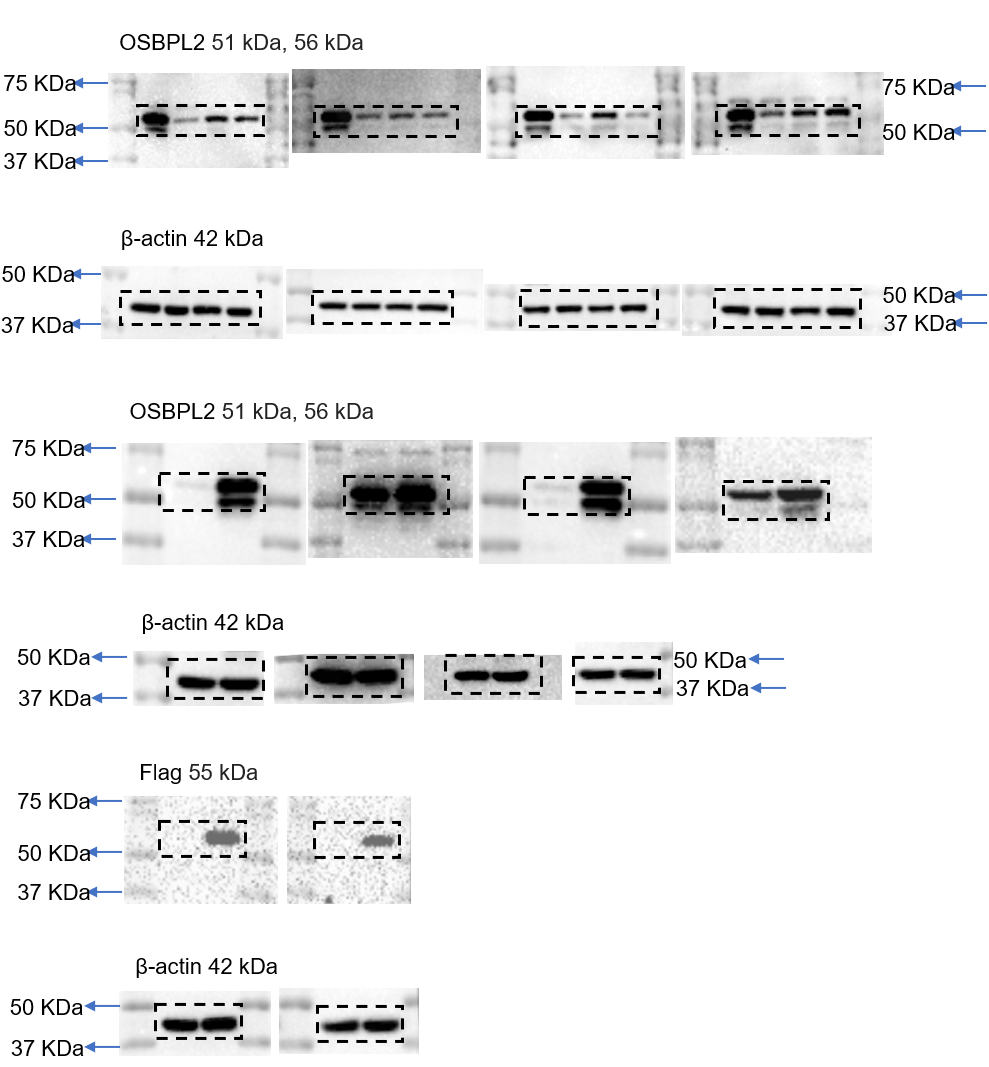


**Supplemental Figure S2** *related to Figure 2*

Immunoblots related to Figure 2, immunoblots of OSBPL2, Flag and β-actin obtained from colorectal cells.


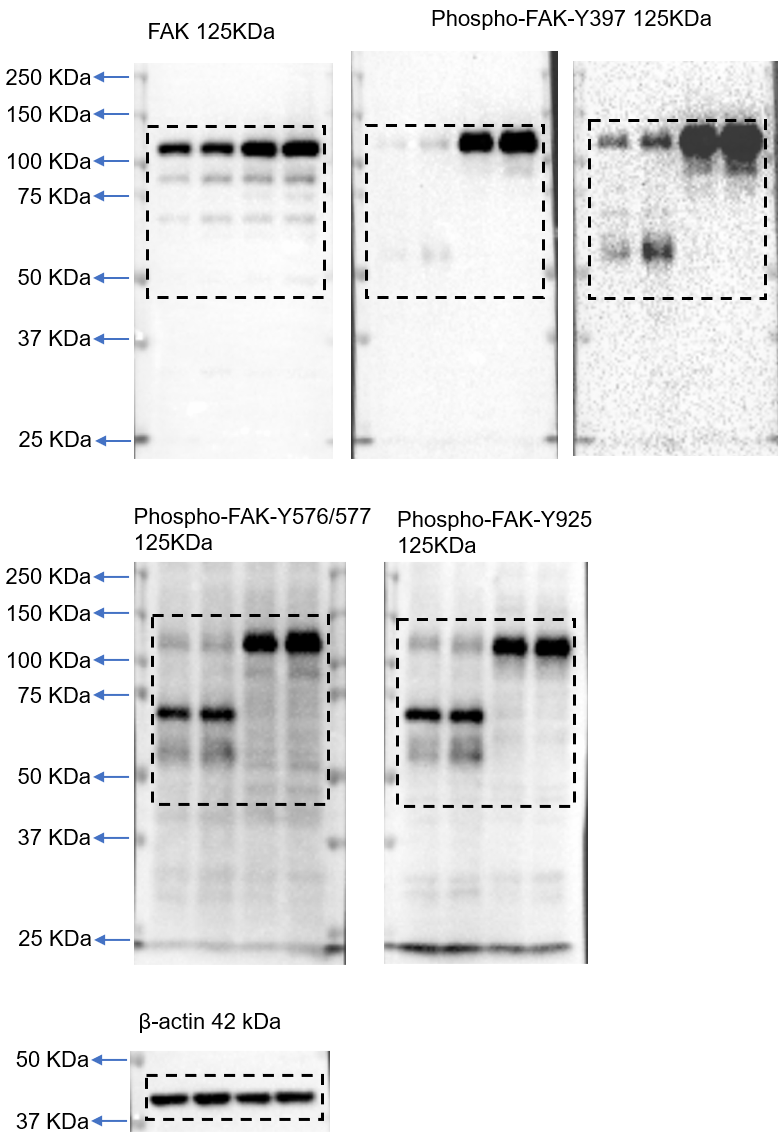


**Supplemental Figure S3** *related to Figure 3*

Immunoblots related to Figure 3, immunoblots of FAK, Phospho-FAK-Y397, Phospho-FAK-Y576/577, Phospho-FAK-Y925 and β-actin obtained from colorectal cells.


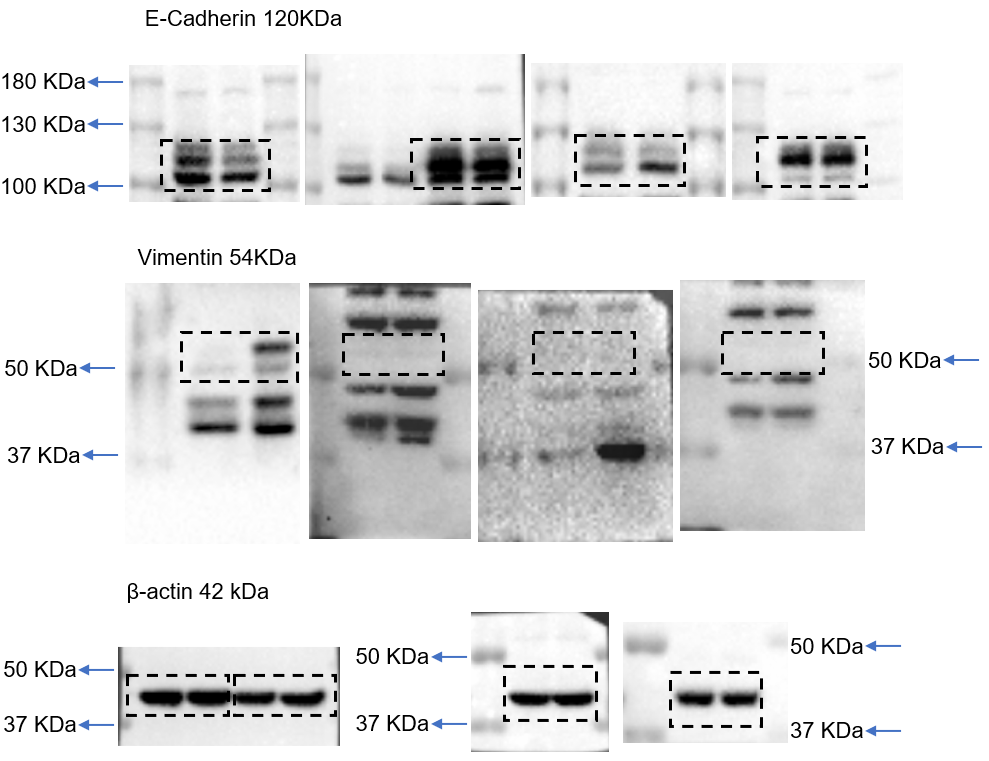


**Supplemental Figure S4** *related to Figure 4*

Immunoblots related to Figure 4, immunoblots of E-Cadherin, Vimentin and β-actin obtained from colorectal cells.


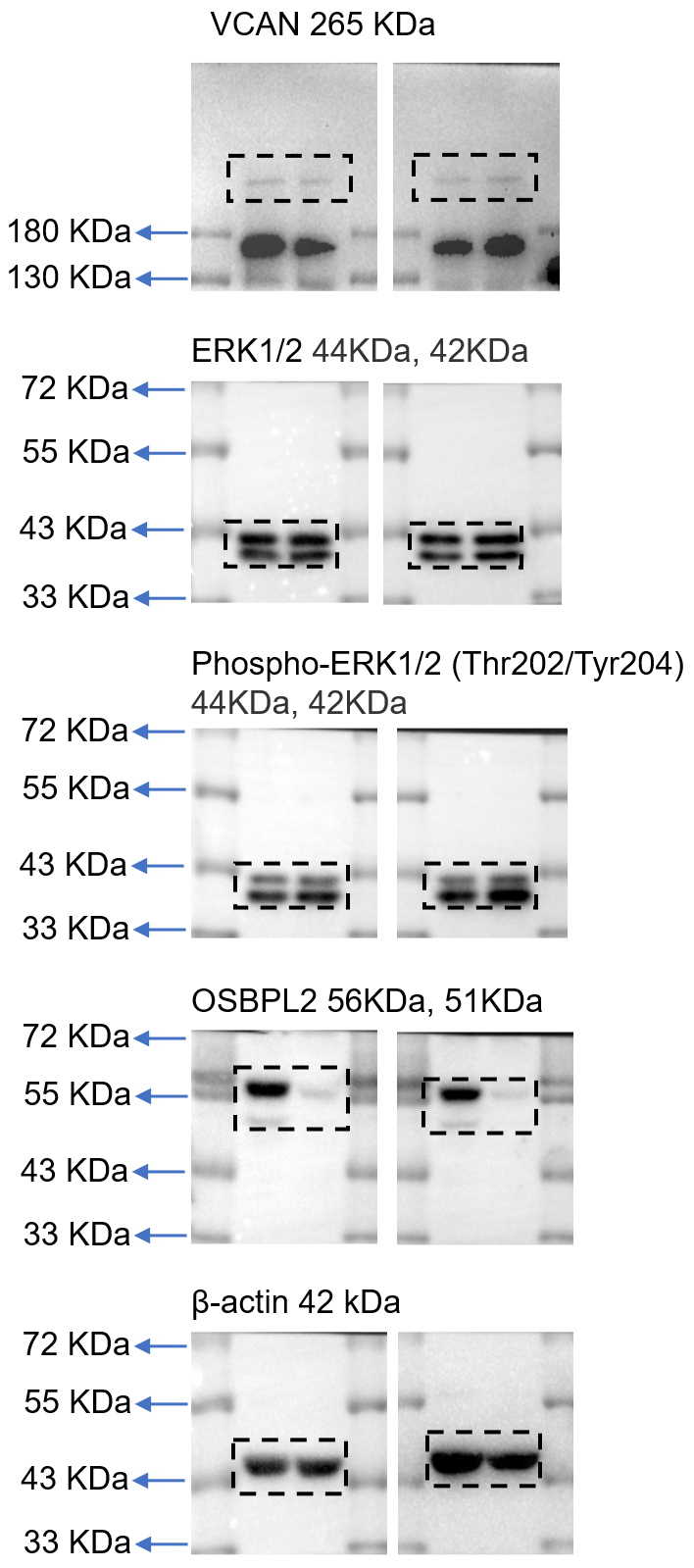


**Supplemental Figure S5** *related to Figure 5M*

Immunoblots related to Figure 5M, immunoblots of VCAN, ERK1/2, Phospho-ERK1/2 (Thr202/Tyr204), OSBPL2 and β-actin obtained from colorectal cells.


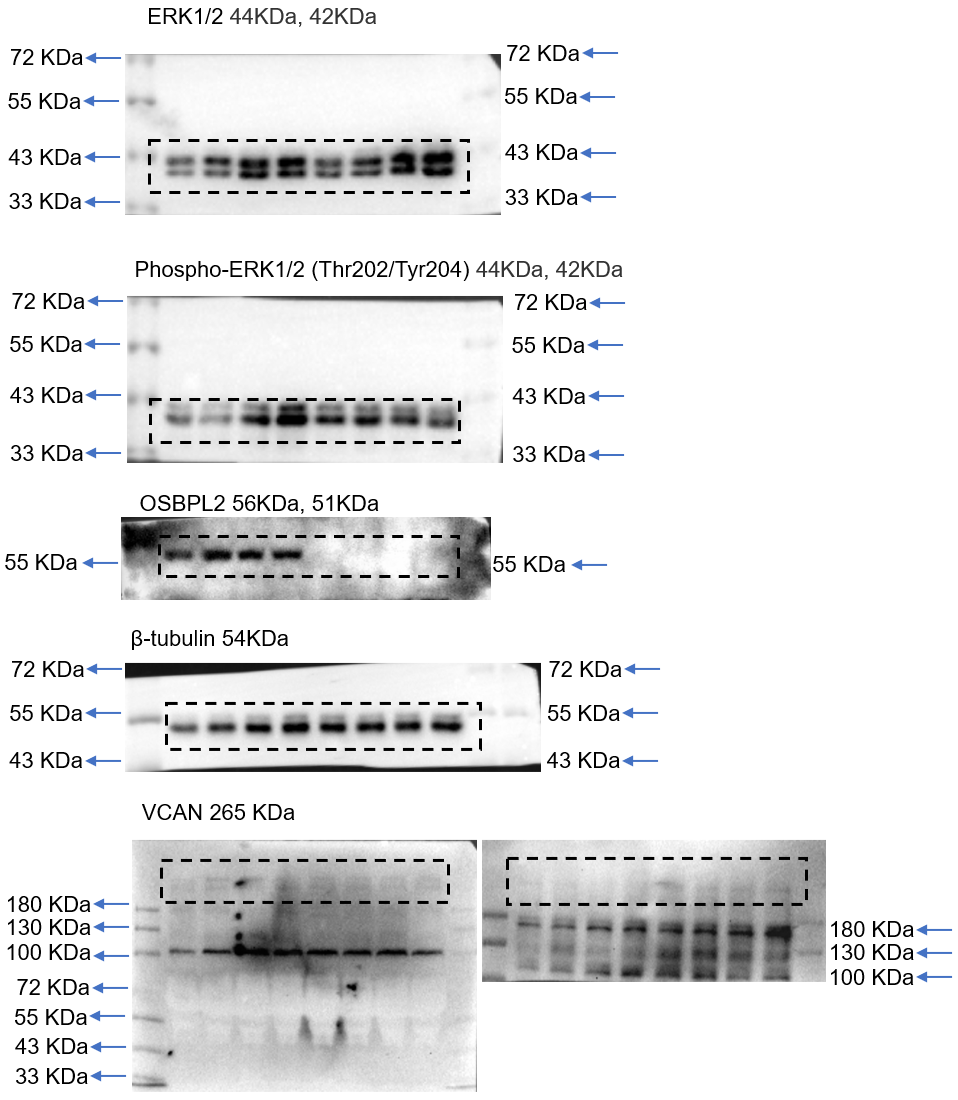


**Supplemental Figure S6** *related to Figure 5O*

Immunoblots related to Figure 5O, immunoblots of ERK1/2, Phospho-ERK1/2 (Thr202/Tyr204), OSBPL2, VCAN and β-tubulin obtained from colorectal cells.


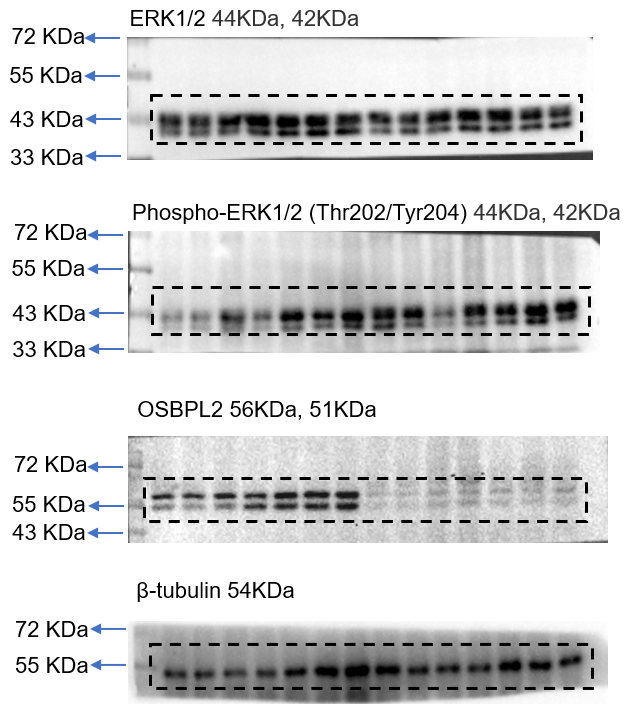


**Supplemental Figure S7** *related to Figure 6T*

Immunoblots related to Figure 6T, immunoblots of ERK1/2, Phospho-ERK1/2 (Thr202/Tyr204), OSBPL2 and β-tubulin obtained from colorectal Xenograft tumor.


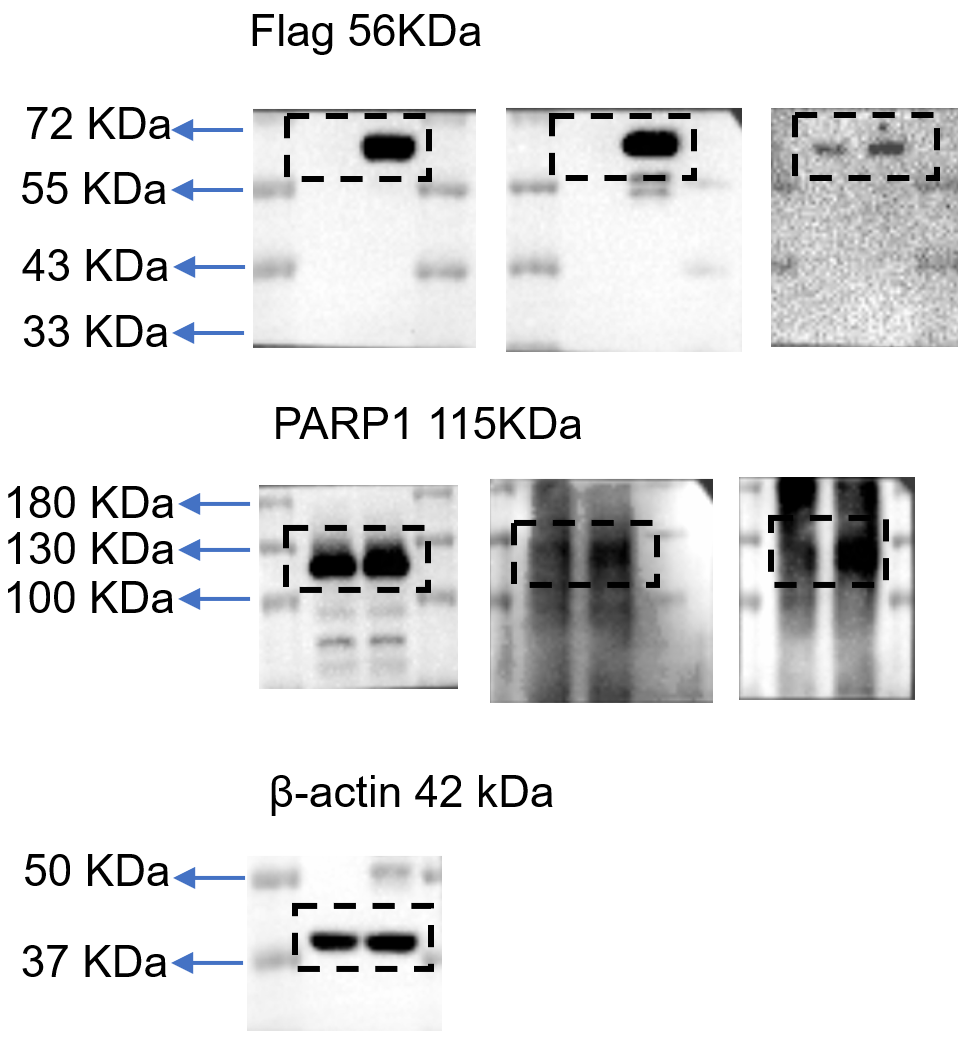


**Supplemental Figure S8** *related to Figure 7C*

Immunoblots related to Figure 7C, immunoblots of Flag, PARP1 and β-actin obtained from colorectal cells.


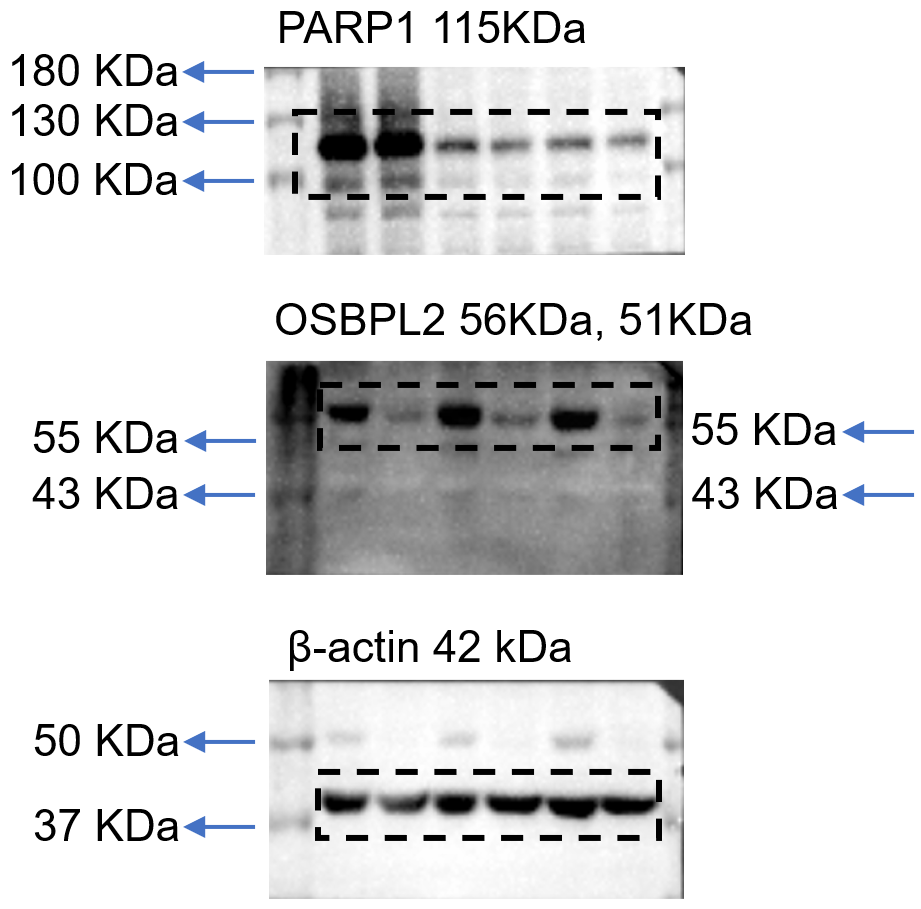


**Supplemental Figure S9** *related to Figure 7M*

Immunoblots related to Figure 7M, immunoblots of PARP1, OSBPL2 and β-actin obtained from colorectal cells.


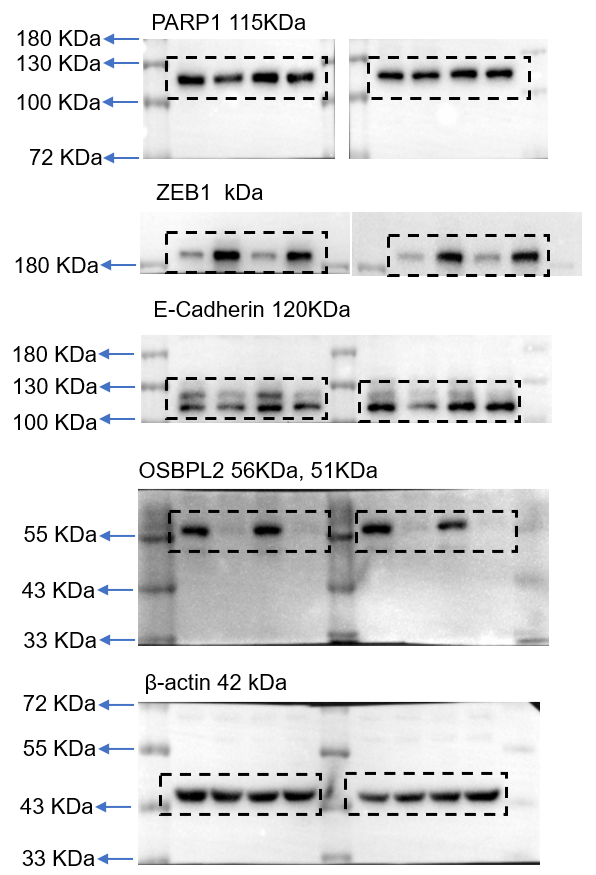


**Supplemental Figure S10** re*lated to Figure 7N*

Immunoblots related to Figure 7N, immunoblots of PARP1, ZEB1, E-Cadherin, OSBPL2 and β-actin obtained from colorectal cells.


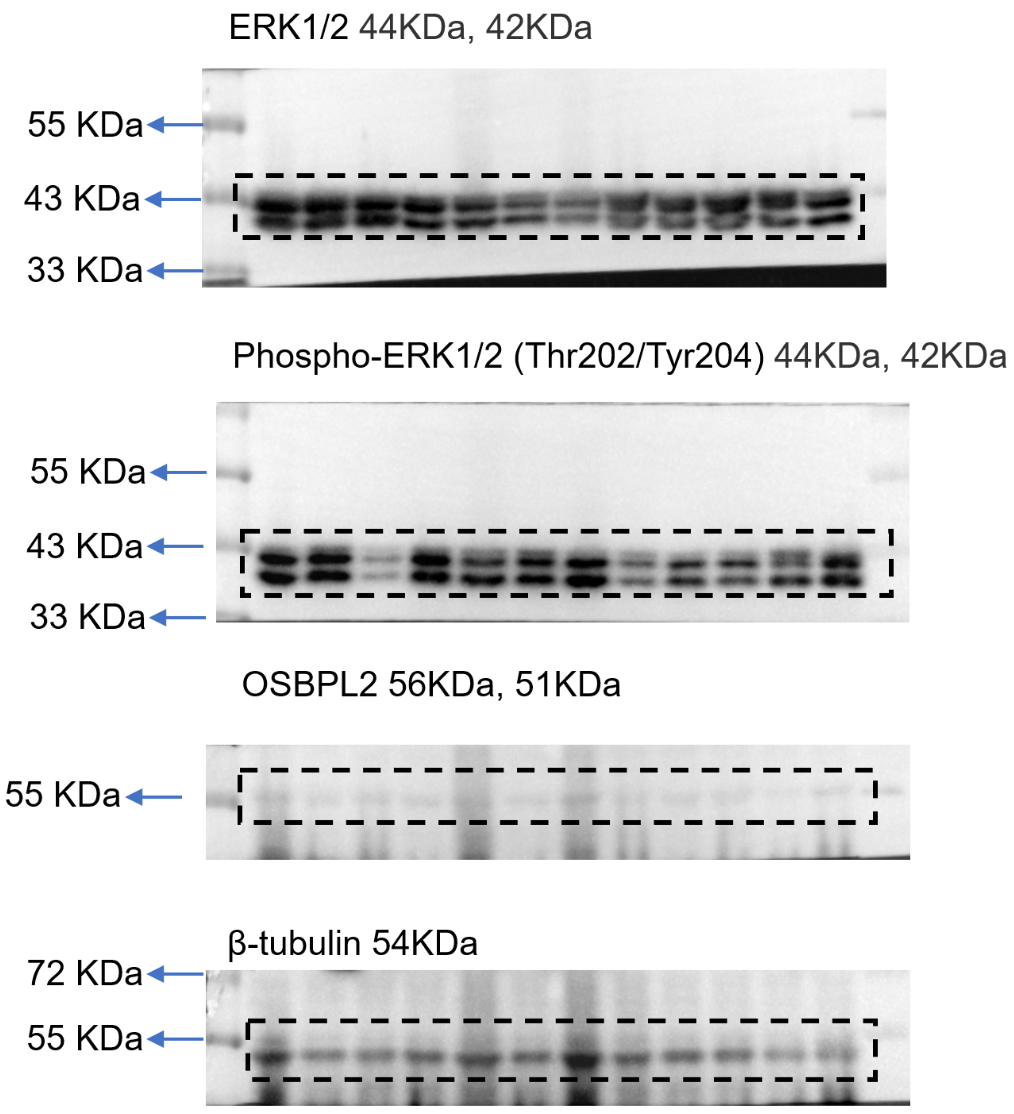


**Supplemental Figure 11** *related to Figure 9*

Immunoblots related to Figure 9, immunoblots of ERK1/2, Phospho-ERK1/2 (Thr202/Tyr204), OSBPL2, and β-tubulin obtained from colorectal cells.


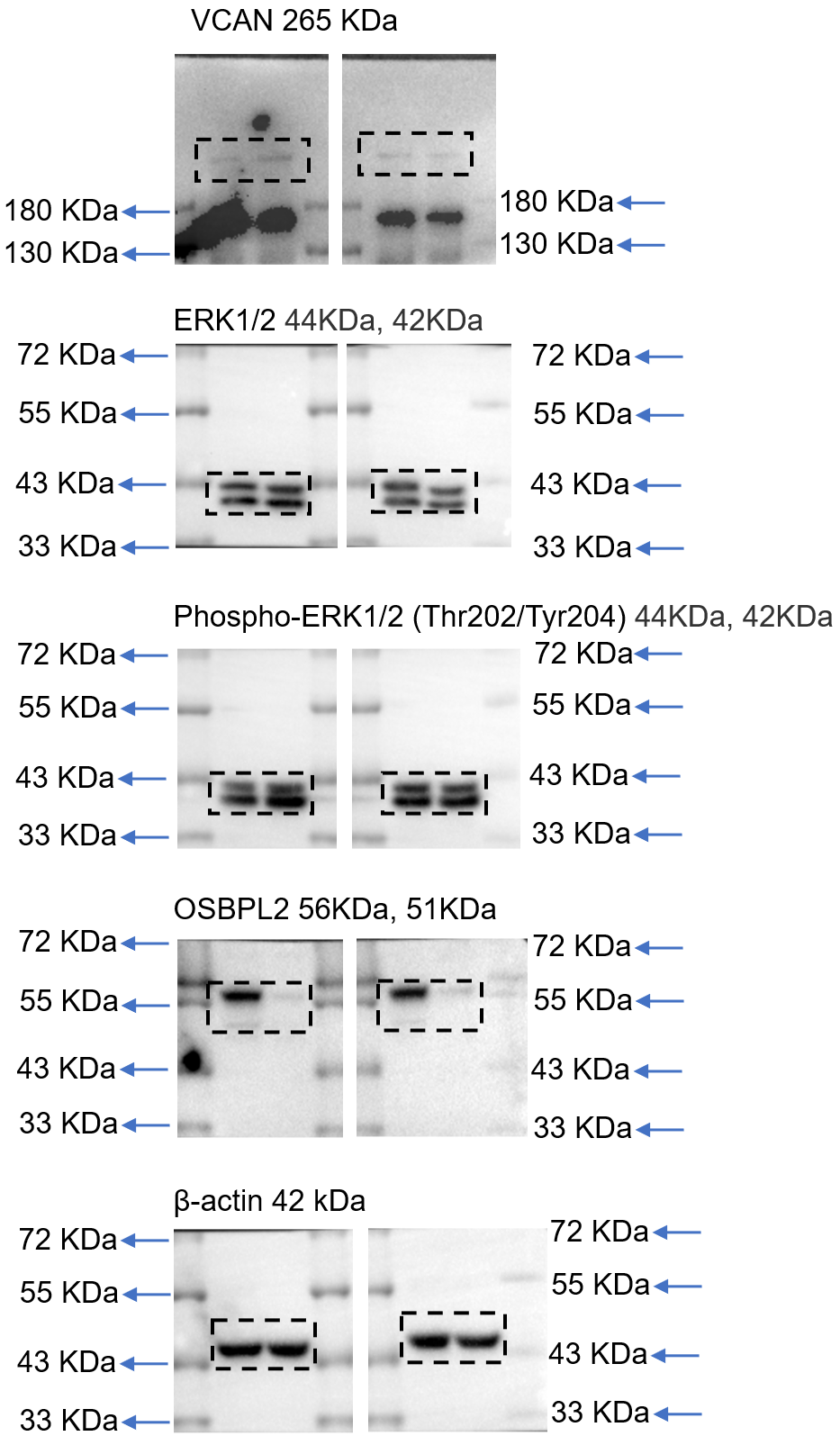


**Supplemental Figure S12** *related to Figure S5F*

Immunoblots related to Figure S5C, immunoblots of VCAN, ERK1/2, Phospho-ERK1/2 (Thr202/Tyr204), OSBPL2, and β-actin obtained from colorectal cells.


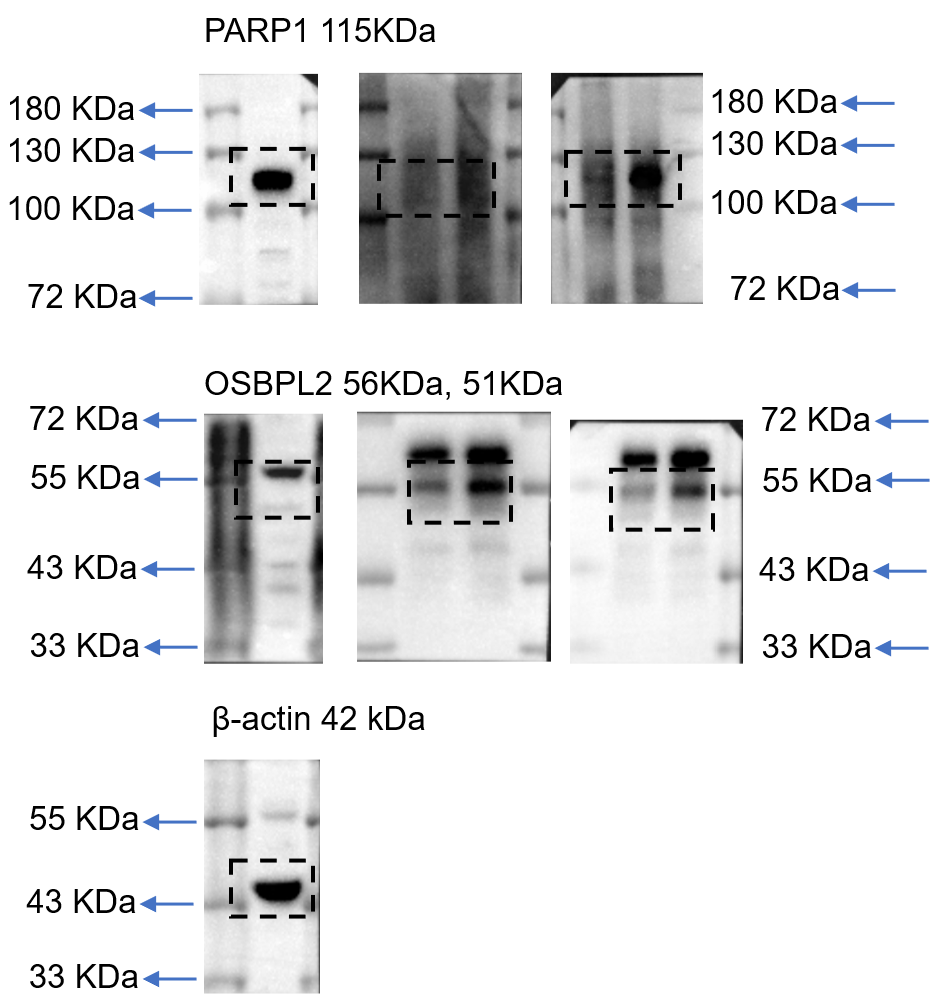


**Supplemental Figure S13** *related to Figure S6A*

Immunoblots related to Figure S6A, immunoblots of PARP1, OSBPL2 and β-actin obtained from colorectal cells.


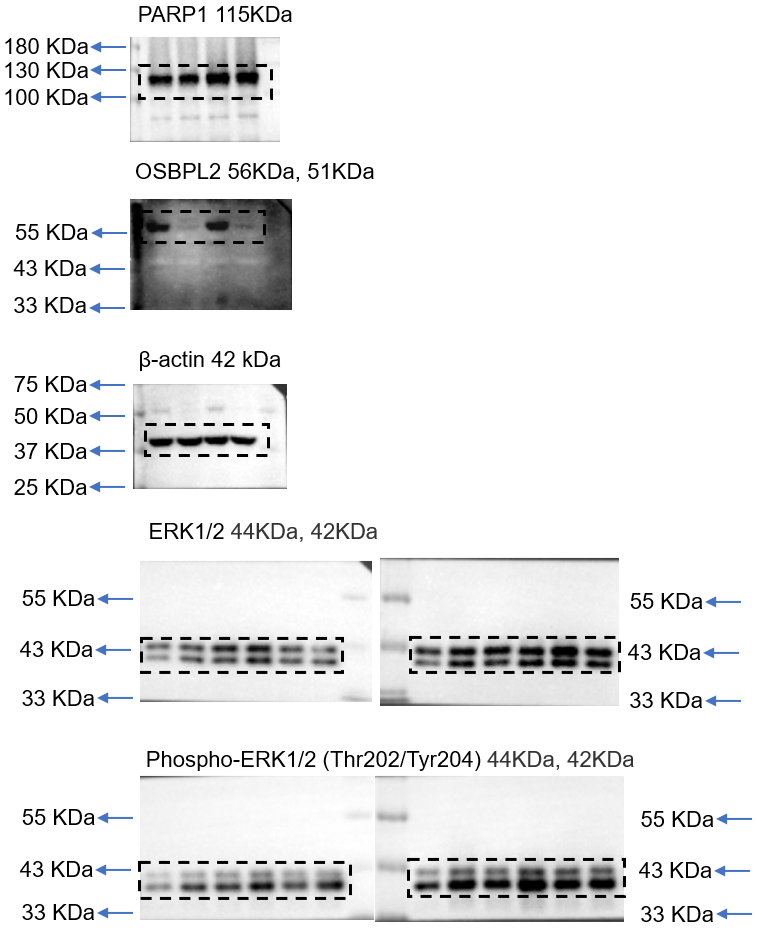


**Supplemental Figure S14** *related to Figure S6C and S6H-I*

Immunoblots related to Figure S6C and S6H-I, immunoblots of PARP1, OSBPL2, ERK1/2, Phospho-ERK1/2 (Thr202/Tyr204), and β-actin obtained from colorectal cells.
